# A single amino acid substitution in a chitinase of the legume *Medicago truncatula* is sufficient to gain Nod-factor hydrolase activity

**DOI:** 10.1098/rsob.160061

**Published:** 2016-07-06

**Authors:** Lan-Yue Zhang, Jie Cai, Ru-Jie Li, Wei Liu, Christian Wagner, Kam-Bo Wong, Zhi-Ping Xie, Christian Staehelin

**Affiliations:** 1 State Key Laboratory of Biocontrol and Guangdong Key Laboratory of Plant Resources, School of Life Sciences, Sun Yat-sen University, East Campus, Guangzhou 510006, People's Republic of China; 2 Chinese University of Hong Kong, Shatin, Hong Kong, People's Republic of China; 3 Shenzhen Research and Development Center of State Key Laboratory of Biocontrol, School of Life Sciences, Sun Yat-sen University, Baoan, Shenzhen, People's Republic of China

**Keywords:** *Medicago truncatula*, chitinase, Nod-factors (lipo-chitooligosaccharides), Nod-factor hydrolase, neofunctionalization

## Abstract

The symbiotic interaction between nitrogen-fixing rhizobia and legumes depends on lipo-chitooligosaccharidic Nod-factors (NFs). The NF hydrolase MtNFH1 of *Medicago truncatula* is a symbiotic enzyme that hydrolytically inactivates NFs with a C16 : 2 acyl chain produced by the microsymbiont *Sinorhizobium meliloti* 1021*.* MtNFH1 is related to class V chitinases (glycoside hydrolase family 18) but lacks chitinase activity. Here, we investigated the substrate specificity of MtNFH1-related proteins. MtCHIT5a and MtCHIT5b of *M. truncatula* as well as LjCHIT5 of *Lotus japonicus* showed chitinase activity, suggesting a role in plant defence. The enzymes failed to hydrolyse NFs from *S. meliloti.* NFs from *Rhizobium leguminosarum* with a C18 : 4 acyl moiety were neither hydrolysed by these chitinases nor by MtNFH1. Construction of chimeric proteins and further amino acid replacements in MtCHIT5b were performed to identify chitinase variants that gained the ability to hydrolyse NFs. A single serine-to-proline substitution was sufficient to convert MtCHIT5b into an NF-cleaving enzyme. MtNFH1 with the corresponding proline-to-serine substitution failed to hydrolyse NFs. These results are in agreement with a substrate-enzyme model that predicts NF cleavage when the C16 : 2 moiety is placed into a distinct fatty acid-binding cleft. Our findings support the view that *MtNFH1* evolved from the ancestral *MtCHIT5b* by gene duplication and subsequent symbiosis-related neofunctionalization.

## 1. Introduction

Chitinases (EC 3.2.1.14) are glycoside hydrolases (GHs) that cleave β-1, 4 glycosidic bonds in chitin (polymer of *N*-acetylglucosamine; poly-GlcNAc) and similar substrates such as glycol chitin. Plants lack chitin but produce chitinolytic enzymes. Most of them appear to play a role in defence against chitin-containing enemies and pathogens [1,2]. For example, various plant chitinases hydrolytically degrade chitin in cell walls of fungi and thus show inhibition effects on growth of fungal mycelia [3,4]. Expression of plant chitinase genes is often induced in response to water-soluble chitin fragments (oligo-GlcNAc). Various endochitinases can release such elicitors from chitin-containing materials, particularly from fungal cell walls. These oligo-GlcNAc elicitors are perceived by corresponding lysin motif (LysM)-containing plant receptors (pattern recognition receptors). Ligand recognition initiates downstream signalling events that culminate in the activation of plant defence responses such as expression of pathogenesis-related (PR) genes. On the other hand, chitinases are able to cleave oligo-GlcNAc and thus have the capacity to reduce the elicitor activity of oligo-GlcNAc [5,6].

Most chitinolytic enzymes listed in the CAZy database can be classified as GH family 18 and 19 enzymes [7]. The reaction mechanism of GH 18 family enzymes is a retaining mechanism (catalysis leads to the retention of the anomeric configuration), whereas GH 19 enzymes use an inverting catalytic mechanism, resulting in anomeric inversion [8]. Plant chitinases are traditionally divided into different classes, and GH family 18 members are subdivided into the classes III and V. While there are numerous reports on class III chitinases [9], only three class V chitinases have been characterized, namely NtChiV from tobacco (*Nicotiana tabacum*) [10–14], AtChiC from *Arabidopsis thaliana* [15,16] and CrChiA from the gymnosperm *Cycas revoluta* [17–20]. Crystal structures for these three enzymes have been solved recently. The proteins consist of a (β/α)_8_ triosephosphate isomerase (TIM) barrel fold containing the catalytic DXDXE motif and a (α + β) insertion domain [13,15,19]. RobpsCRA, a lectin of the legume tree *Robinia pseudoacacia* with sequence similarities to class V chitinases, possesses a similar structure [21,22].

Owing to structural similarities to oligo-GlcNAc, various plant chitinases are able to hydrolyse nodulation factors (Nod-factors, NFs) [23–28]. NFs are bacterial signal molecules produced by nitrogen-fixing rhizobia that establish a nodule symbiosis with leguminous plants such as *Medicago truncatula* and *Lotus japonicus*. NFs consist of an oligo-GlcNAc backbone, which is acylated at the non-reducing end. NFs may contain additional groups at both ends of the oligo-GlcNAc moiety, depending on the strain analysed [29]. For example, *Sinorhizobium* (*Ensifer*) *meliloti* produces pentameric (V) and tetrameric (IV) NFs (NodSm factors) that are mainly *N*-acylated by a C16 : 2 fatty acid (2E,9Z-hexadecadienoic acid) on the non-reducing end. The sugar moiety is sulfated (S) at the reducing end and partially *O*-acetylated (Ac) at the non-reducing end [30,31]. NFs are specifically perceived by LysM-type NF receptors of the host plant to trigger symbiosis-related responses such as expression of symbiosis-related genes [32,33]. Remarkably, activities or transcript levels of certain chitinases or chitinase-related hydrolases are upregulated in various host legumes when roots are inoculated with rhizobia or treated with purified NFs, suggesting a role of these enzymes in inactivation of excess amounts of NFs [25,34–39]. Purified cleavage products of NFs (lipo-trisaccharides and lipo-disaccharides) showed only poor biological activity, indicating that hydrolytic NF degradation results in signal inactivation [24,40].

Based on previous studies [34,37], we have recently identified and characterized MtNFH1 (*M. truncatula* Nod-factor hydrolase 1), an extracellular enzyme that degrades NFs of the microsymbiont *S. meliloti*. The lipo-disaccharide NodSm-II(C16 : 2) was released from pentasaccharidic NodSm-V(C16 : 2, S) and tetrasaccharidic NodSm-IV(C16 : 2, S). Formation of the *O*-acetylated lipo-disaccharide NodSm-II(C16 : 2, Ac) was observed when NodSm-IV(C16 : 2, Ac, S) was used as a substrate [38]. Increased levels of *MtNFH1* transcripts were found in roots (including root hairs) when *M. truncatula* plants were inoculated with *S. meliloti* or treated with NFs [37,39,41]. The *MtNFH1* gene, formerly named *chit5* [37], was originally annotated as a putative class V chitinase. However, enzyme tests with purified MtNFH1 indicated that the protein degrades neither chitin nor oligo-GlcNAc [38]. Hence, MtNFH1 represents a novel GH that specifically cleaves oligo-GlcNAc with a fatty acid chain. The three-dimensional structure of MtNFH1 was modelled, using the class V chitinases NtChiV [13] and AtChiC [15] as structural templates. Substrate-docking simulation with *S. meliloti* NFs suggested that two loops in MtNFH1 (loops A and B) form a binding cleft for the fatty acid moiety of the NF substrate [38].

In this article, we report on the enzyme properties of legume proteins with sequence similarities to MtNFH1. Two enzymes of *M. truncatula* (MtCHIT5a, MtCHIT5b; *M. truncatula* class V chitinases a and b) and a *L. japonicus* homologue (LjCHIT5; *L. japonicus* class V chitinase) showed chitinase activity but failed to degrade *S. meliloti* NFs. Construction of chimeric proteins and further amino acid replacements in the loops A and B of MtCHIT5b were performed to identify protein variants that gained the ability to hydrolyse NFs. The obtained results are in agreement with a substrate-enzyme model that predicts NF cleavage when the C16 : 2 moiety is placed in a distinct fatty acid-binding cleft.

## 2. Material and methods

### 2.1. Biological material

Roots and leaves from four-week-old *M. truncatula* (ecotype R108-1) and six-week-old *L. japonicus* (ecotype Miyakojima MG-20) plants were used for isolation of genomic DNA and RNA. For gene expression analysis, plants were inoculated with *Fusarium oxysporum* f. sp. *cubense* race 4 (originally isolated from banana). The fungus was kindly provided by Dr Jianghui Xie (Chinese Academy of Tropical Agricultural Sciences, Zhanjiang, China). *Trichoderma viride* GIM3.141 obtained from the Guangdong Culture Collection Center (Guangzhou, China) served as test fungus to study effects of recombinant proteins on fungal growth. *Escherichia coli* strain DH5α (Invitrogen, Carlsbad, CA) carrying the plasmids pET28b (6xHis tag) or pET32a (6xHis and Trx tags) from Novagen/Merck (Darmstadt, Germany) was used for gene cloning and strain BL21 (DE3) (Novagen/Merck) for protein expression.

### 2.2. Gene cloning and plasmid construction

For PCR-based cloning of *MtCRA2* and *MtCHIT5b* (accession numbers KU041647 and KU041646), genomic DNA of four-week-old *M. truncatula* (ecotype R108) was isolated according to the cetyltrimethylammonium bromide method [42]. RNA from roots of six-week-old *L. japonicus* (ecotype Miyakojima MG-20) isolated with an RNA extraction kit (Tiangen, Beijing, China) was used for synthesis of cDNA, which served as template for PCR-based cloning of *LjCHIT5* (accession number KU041645). DNA sequences encoding proteins without predicted signal peptides were then cloned into the expression vector pET28b. Plasmids encoding chimeric proteins and proteins with amino acid substitutions were constructed by overlapping extension PCR techniques. The resulting amplicons were then inserted into the expression vectors pET28b or pET32a. All constructed plasmids were verified by sequencing. Plasmids and primers used in this study are listed in electronic supplementary material (tables S6 and S7).

### 2.3. Recombinant proteins

*Escherichia coli* BL21 (DE3) cells carrying a given pET28b or pET32a derivative were grown in Luria–Bertani (LB) medium containing 50 µg ml^−1^ kanamycin or 100 µg ml^−1^ ampicillin at 37°C. Protein expression was stimulated by addition of isopropyl-β-d-thiogalactopyranoside to reach a final concentration of 0.4 mM. Cultures were then kept on a shaker (220 r.p.m.) at 18°C for 20 h. Proteins were purified by nickel affinity chromatography according to the protocol supplied by the manufacturer of the Ni–NTA resin beads (Qiagen, Hilden, Germany). Native conditions were used for purification to obtain functional proteins for subsequent enzyme assays.

### 2.4. Protein analysis

Proteins were characterized by 12% sodium dodecyl sulfate–polyacrylamide gel electrophoresis (SDS–PAGE). The amounts of proteins were determined following the Bradford method with bovine serum albumin as standard [43]. Protein gels were either stained with Coomassie brilliant blue R-250 or used for Western blot analysis with an anti-MtNFH1 antibody [38]. Goat–anti-rabbit IgG antiserum coupled to horseradish peroxidase was used as second antibody, and blots were developed with 3,3′-diamino-benzidine (Boster, Wuhan, China).

### 2.5. Purification of Nod-factors

Pentameric NodSm-V(C16 : 2, S), tetrameric NodSm-IV(C16 : 2, S) and *O*-acetylated NodSm-IV(C16 : 2, Ac, S) were HPLC-purified from *S. meliloti* strain 1021 (pEK327) as described previously [31,38]. NFs from *R. leguminosarum* bv*. viciae*, namely NodRlv-V(C18 : 4, Ac) and NodRlv-IV(C18 : 4, Ac), were isolated and HPLC-purified from the overproducing strain *R. leguminosarum* bv*. viciae* strain RBL 5799 [44] as reported before [28].

### 2.6. Enzyme assays with chitinous substrates

Non-modified chitin oligosaccharides [(GlcNAc)*_n_*, *n* = 2–5] were provided by Seikagaku Kogyo Co. (Tokyo, Japan). Hexameric (GlcNAc)_6_ was purchased from Elicityl (Crolles, France). Enzyme reactions were performed with 5.8 mM (GlcNAc)_6_ and different enzymes (4.5 µg ml^−1^) dissolved in 25 mM sodium acetate buffer (pH 5.0) at 37°C in a 50 µl test volume. Each reaction mixture was then directly loaded onto the HPLC amino column (TSK-GEL Amino-80 column 4.6 × 250 mm; Tosoh, Tokyo, Japan). 70% (v/v) acetonitrile/water was used as mobile phase, and the flow rate was 0.7 ml min^−1^. Peaks were detected with a UV detector at 220 nm. For MtCHIT5b, kinetic parameters were determined with (GlcNAc)_5_ and (GlcNAc)_6_ as substrates. GraphPad Prism v. 5.00 (GraphPad Software, San Diego, CA) was used for calculation of *K*_m_ and *k*_cat_ values (electronic supplementary material, table S2).

Assays with polymeric substrates were performed at 37°C with specified amounts of enzymes in 25 mM sodium acetate buffer (pH 5.0). Colloidal chitin was chemically synthesized from chitosan (Sigma-Aldrich, St Louis, MO) incubated with acetic anhydride [45]. Recombinant *N*-acetylglucosaminidase from *M. truncatula* was used to hydrolyse released oligosaccharides into monomeric GlcNAc. Quantification of GlcNAc was photometrically performed with *p*-dimethylaminobenzaldehyde (Ehrlich's reagent) [46]. Glycol chitin was obtained by acetylation of glycol chitosan with acetic anhydride (Sigma-Aldrich) as reported [45]. Hydrolysis of glycol chitin was quantified by measuring the reducing end sugars with the Lever assay [47]. The substrate CM–chitin–RBV (Carboxymethyl–chitin–Remazol brilliant violet 5R) was purchased from Loewe Biochemica (Sauerlach, Germany), and the photometer assay was performed according to a previously established procedure [48].

### 2.7. Enzyme assays with Nod-factors

Defined amounts of purified proteins and NFs were incubated in 25 mM sodium acetate buffer (pH 5.0) at 37°C for an indicated time period. The NF substrates and acylated cleavage products were extracted by an equal volume of *n*-butanol and dried in a rotary evaporator. Samples were taken up by 1 µl DMSO followed by 60 µl 50% acetonitrile and loaded on a reverse phase HPLC column (Nova Pak C18, 3.9 × 150 mm, particle size 4 mm; Waters). Samples were separated under isocratic conditions at a flow rate of 1 ml min^−1^. NFs from strain 1021 (pEK327) and formed acylated degradation products, i.e. NodSm-II(C16 : 2) or NodSm-II(C16 : 2, Ac) were fractioned using 36% (v/v) acetonitrile/water and 40 mM ammonium acetate as mobile phase. Molecules were photometrically detected at 220 nm [24]. For samples with NFs of strain RBL 5799, 36% (v/v) acetonitrile/water was used as mobile phase, and molecules were detected at 304 nm [28]. Commercial hen egg-white lysozyme hydrolysed the NFs of RBL 5799 and served as a reference enzyme to produce the cleavage product NodRlv-III(C18 : 4, Ac). To measure activities and kinetic parameters, the incubation time was varied in order that the percentage of degradation was less than 25% of the initial substrate. The *K*_m_ and *k*_cat_ values were determined, using GraphPad Prism v. 5.00.

### 2.8. Lysozyme assay and fungal growth inhibition test

Lyophilized *Micrococcus lysodeikticus* from Sigma-Aldrich was used for the lysozyme assay with recombinant proteins dissolved in 25 mM sodium acetate buffer (pH 5.0). The decrease in turbidity of the cell suspension was photometrically measured at 645 nm [49]. Inhibition effects of recombinant proteins on growth of the fungus *T. viride* grown on potato dextrose agar were examined at 27°C as described previously [3].

### 2.9. Mass spectrometric analysis

Where indicated, samples (lipo-disaccharides; non-modified oligo-GlcNAc) were subjected to analysis by an Ultraflex III MALDI–TOF/TOF mass spectrometer (Bruker Daltonics, Billerica, MA). The instrument was operating in a positive-ion mode and equipped with a smartbeam ultraviolet laser (*λ*, 355 nm). All samples (typically 2 µl) were mixed (1 : 1 ratio) with the 2,5-dihydroxybenzoic acid matrix (saturated in acetonitrile). The sample-matrix mixture was dried on a conventional 49-place stainless steel target. Observed ion masses of lipo-disaccharides corresponded to [M + H]^+^ owing to protonation and [M + K]^+^ owing to the formation of potassium adducts (electronic supplementary material, table S3 and figure S9).

### 2.10. Expression analysis of *MtCHIT5b* and *LjCHIT5* by quantitative reverse transcription PCR

For expression analysis of *MtCHIT5b* and *LjCHIT5*, 30-day-old *M. truncatula* and 40-day-old *L. japonicus* plants were inoculated with a suspension of *F. oxysporum* f. sp. *cubense* race 4 (in 10 mM MgSO_4_; 1.6 × 10^6^ spores per ml). RNA from roots and leaves of inoculated and mock-inoculated plants was isolated, using an RNA extraction kit according to the instructions of the manufacturer (Tianze, Guangzhou, China). qRT-PCRs with synthesized cDNA and gene-specific primers for *MtCHIT5b* and *LjCHIT5* were performed by using the LightCycler 480 SYBR Green I Master Mix in a LightCycler 480 System (Roche Diagnostics, Mannheim, Germany). *Mtactin-97* and *Ljubiquitin* served as reference genes. Used primers are listed in electronic supplementary material (table S7). All reactions were done in triplicates. The threshold cycle (*C*_T_) values, which are the cycle numbers required for the SYBR green I fluorescent signal to cross the threshold value, were calculated using the LightCycler v. 480 software.

### 2.11. Homology modelling and substrate-docking simulation

The crystal structures of the class V chitinases of tobacco [13] (NtChiV; Protein Data Bank (PDB) codes 3ALF and 3ALG) and *A. thaliana* [15] (AtChiC; PDB code: 3AQU) served as templates for homology modelling of MtNFH1 and MtCHIT5b. The structure-based sequence alignment (electronic supplementary material, figure S10) was obtained interactively by using the program Swiss-PdbViewer (http://spdbv.vital-it.ch/). Models for MtNFH1 and MtCHIT5b were created with the program Modeller [50]. Substrate-docking simulation of the substrate NodSm-V(C16 : 2, S) to MtNFH1 was performed as described previously [38].

## 3. Results

### 3.1. Enzyme activities of MtNFH1-related proteins

**Figure 1.**
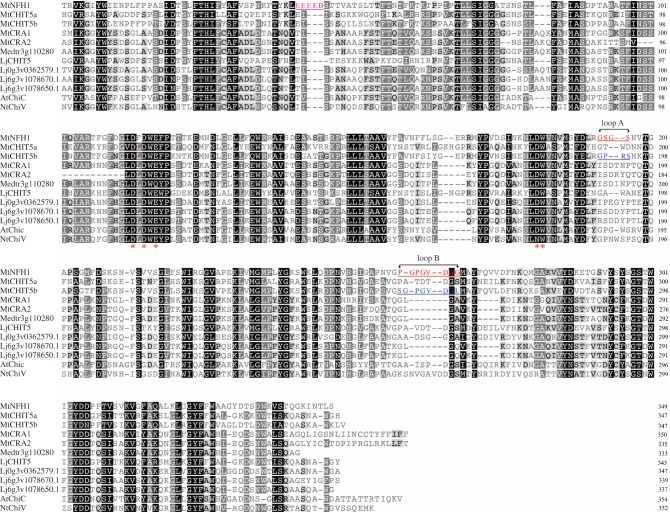
Alignment of amino acid sequences of MtNFH1 and MtNFH1-related sequences deduced from nucleotide sequences of *M. truncatula* and *L. japonicus.* Reference sequences of AtChiC and NtChiV were included into the alignment. The alignment was performed with Geneious software (http://www.geneious.com). N-terminal residues (including predicted signal peptides) are not shown in the alignment. Red asterisks indicate predicted residues required for enzyme activity (the catalytic DxDxE motif is conserved in GH 18 family enzymes). Amino acid residues of loop A and loop B in MtNHF1 and MtCHIT5b are marked in red and blue, respectively. The unique EEEED motif in MtNFH1 is marked in purple. Identical amino acid residues are shown on a black background, homologous residues on a grey background and dashes indicate gaps. Sequences: (i) *M. truncatula* ecotype R108-1: MtNFH1 (accession no. KC833515), MtCHIT5a (KC833513), MtCHIT5b (KU041646), MtCRA1 (KC833514), MtCRA2 (KU041647) and Medtr3g110280 (XP_013462049.1); (ii) *L. japonicus* ecotype Miyakojima MG-20: LjCHIT5 (KU041645), Lj0g3v362579.1, Lj6g3v1078670.1 (AFK36566.1) and Lj6g3v1078650.1 (AFK36566.1); (iii) reference sequences: AtChiC of *A. thaliana* (NP_193716, 3AQU) and NtChiV of *N. tabacum* (CAA55128, CAA54373, 3ALF).

To characterize proteins related to the previously identified NF-cleaving enzyme MtNFH1, we performed a study with class V chitinase family proteins of *M. truncatula* and a homologue from the legume *L. japonicus*. An amino acid sequence alignment of predicted MtNFH1-related proteins is shown in figure 1 and a corresponding phylogenetic tree in the electronic supplementary material (figure S1). In total, six proteins were analysed, namely MtNFH1 [38], MtCHIT5a [38] (*M. truncatula* class V chitinase a; KC833513), MtCHIT5b (*M. truncatula* class V chitinase b; KU041646), MtCRA1 (*M. truncatula* chitinase-related agglutinin 1; previously named MtCRA [38]), MtCRA2 (*M. truncatula* chitinase-related agglutinin 2; KU041647) and LjCHIT5 (*L. japonicus* class V chitinase; KU041645). The MtCHIT5b sequence is most similar to MtNFH1 (77% amino acid identity in sequences without predicted signal peptide). The corresponding genes are located in tandem on chromosome 4 of *M. truncatula* (electronic supplementary material, figure S2). LjCHIT5 shows the closest relationship with MtCHIT5a. An overview of the studied proteins with corresponding accession numbers, predicted biochemical properties and summarized expression data is shown in electronic supplementary material, table S1. Expression of the corresponding genes was previously analysed with the Affymetrix GeneChip technology, and obtained data are hosted by the gene expression atlas web servers for *M. truncatula* [41] and *L. japonicus* [51]. Elevated transcript levels of *MtNFH1* in response to *S. meliloti* inoculation and NFs were found in roots [37] and root hairs [39] as measured by quantitative reverse transcription PCR (qRT-PCR) [37] and GeneChip hybridizations [39,41]. In addition, we measured the transcript levels of *MtCHIT5b* and *LjCHIT5* by qRT-PCR. The results show increased accumulation of transcripts in response to inoculation with the fungal pathogen *F. oxysporum* (electronic supplementary material, figure S3).

Nucleotide sequences of the *MtNFH1*-related genes (without the predicted N-terminal signal peptide sequences) were cloned into expression plasmids and recombinant proteins with an N-terminal 6xHis tag were purified from *E. coli* cells. SDS–PAGE gels of purified proteins showed bands that corresponded to the expected molecular weight of the recombinant proteins. Rabbit antibodies against MtNFH1 [38] cross-reacted with the recombinant proteins, confirming successful purification (electronic supplementary material, figure S4).

**Figure 2.**
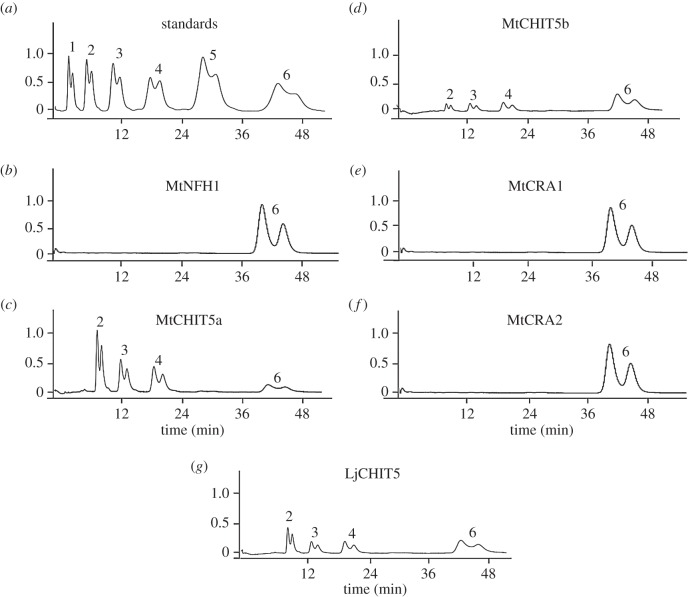
Activity test with recombinant proteins and (GlcNAc)_6_. HPLC analysis of (GlcNAc)_6_ and degradation products was performed after incubation (37°C) with indicated proteins. The reaction mixtures (0.05 ml) containing 5.8 mM (GlcNAc)_6_ and recombinant proteins were separated on a TSK-GEL Amino-80 column. Oligo-GlcNAc molecules were separated into anomers (double peaks). (*a*) Oligo-GlcNAc standards (10 nmol) with degree of polymerization (dp) = 1–6; (*b*) (GlcNAc)_6_ incubated with MtNFH1 (5.0 µg ml^−1^) for 4 h; (*c*) (GlcNAc)_6_ incubated with MtCHIT5a (5.0 µg ml^−1^) for 30 min; (*d*) (GlcNAc)_6_ incubated with MtCHIT5b (5.0 µg ml^−1^) for 30 min; (*e*) (GlcNAc)_6_ incubated with MtCRA1 (5.0 µg ml^−1^) for 30 min; (*f*) (GlcNAc)_6_ incubated with MtCRA2 (5.0 µg ml^−1^) for 30 min; and (*g*) (GlcNAc)_6_ incubated with LjCHIT5 (4.5 µg ml^−1^) for 30 min.

The proteins were then used for enzyme assays with (GlcNAc)_6_ (chitin hexaose) as substrate. In contrast to MtNFH1 [38], MtCHIT5a, MtCHIT5b and LjCHIT5 could hydrolyse (GlcNAc)_6_ into (GlcNAc)_4_ and (GlcNAc)_2_ or 2 (GlcNAc)_3_ molecules (figure 2). The structures of the degradation products produced by MtCHIT5b were confirmed by MALDI–TOF mass spectrometry analysis (electronic supplementary material, figure S5). Kinetic data for MtCHITb were acquired, using various concentrations of (GlcNAc)_6_ and (GlcNAc)_5_. The results show Michaelis–Menten constants for these substrates in the millimolar range (electronic supplementary material, table S2). In accordance with previous studies on the tobacco class V chitinase NtChiV [13,14], non-functional protein variants of MtCHIT5a and LjCHIT5 were obtained when amino acid residues essential for catalytic activity were substituted by alanine (D148A and W364A in MtCHIT5a; D146A and W361A in LjCHIT5; numbering of residues according to the full-length coding sequences; accession numbers KC833513 and KU041645). The RobpsCRA-related proteins MtCRA1 and MtCRA2 did not degrade (GlcNAc)_6_.

Purified NFs and various chitinase substrates were used to further characterize the substrate specificity of the recombinant proteins. MtNFH1 specifically released lipo-disaccharides from *S. meliloti* NFs carrying a C16 : 2 acyl chain as reported previously [38]. However, NFs from *R. leguminosarum* bv. *viciae* with a C18 : 4 acyl chain [44] were not cleaved by MtNFH1. In addition to (GlcNAc)_6_, MtCHIT5a, MtCHIT5b and LjCHIT5 hydrolysed various chitinous substrates, namely colloidal chitin, glycol chitin and the chromogenic substrate CM–chitin–RBV. However, these three chitinases did not cleave the purified NFs of *S. meliloti* and *R. leguminosarum* bv. *viciae*. Furthermore, the proteins did not show lysozyme activity as assayed with *M. lysodeikticus* cells. The MtCRA1 and MtCRA2 proteins failed to hydrolyse any of the tested substrates (table 1).

**Table 1.** Activities of 6xHis-tagged recombinant proteins with various substrates.

substrate	enzyme activity (nkat mg^−1^)^a^						
	MtNFH1^b^	MtCHIT5a	MtCHIT5b	MtCRA1	MtCRA2	LjCHIT5	assay
NodSm-V(C16 : 2, S)	154.1 ± 28.2	ND	ND	ND	ND	ND	reverse-phase HPLC analysis (C18 column)
NodSm-IV(C16 : 2, S)	116.7 ± 3.4	ND	ND	ND	ND	ND	
NodRlv-V(C18 : 4, Ac)	ND	ND	ND	ND	ND	ND	
NodRlv-IV(C18 : 4, Ac)	ND	ND	ND	ND	ND	ND	
(GlcNAc)_6_	ND	368.2 ± 7.5^c^	138.4 ± 19.3^c^	ND	ND	375.2 ± 5.1^c^	reverse-phase HPLC analysis (amino column)
colloidal chitin	ND	0.013 ± 0.009	0.007 ± 0.006	ND	ND	0.046 ± 0.015	Ehrlich's reagent
glycol chitin	ND	12.2 ± 0.3	4.4 ± 0.3	ND	ND	14.2 ± 0.7	Lever assay
CM–chitin–RBV	ND	0.80 ± 0.12^d^	1.44 ± 0.02	ND	ND	0.75 ± 0.06	colorimetric assay
*M*. *lysodeikticus* cells	ND	ND	ND	ND	ND	ND	lysozyme assay

^a^Enzyme assays were performed at 37°C with a substrate concentration of 150 µM for NodSm-V(C16 : 2, S) and NodSm-IV(C16 : 2, S) from *S. meliloti*, 50 µM for NodRlv-V(C18 : 4, Ac) or NodRlv-IV(C18 : 4, Ac) from *R. leguminosarum* bv. *viciae*, 3.6 mM for (GlcNAc)_6_, approximately 10 mg ml^−1^ for colloidal chitin, 20 mg ml^−1^ for glycolchitin, 0.9 mg ml^−1^ for CM–chitin–RBV, and 0.45 mg ml^−1^ for *M. lysodeikticus* cells. Data indicate means ± s.d. from at least three independently purified enzyme preparations. ND, not degraded.

^b^Similar results were obtained previously [38].

^c^Cleavage into (GlcNAc)_4_ and (GlcNAc)_2_ or 2 (GlcNAc)_3_.

^d^Enzyme activity expressed as ΔA_550_ **^.^** (mg protein)^−1^ s^−1^.

MtCHIT5a, MtCHIT5b and LjCHIT5 were also tested for their capacity to inhibit hyphal growth of the fungus *T. viride* in an agar-plate bioassay. In contrast to MtNFH1 [38], MtCHIT5a and LjCHIT5 showed fungal growth inhibition activity. Reduced growth of the mycelium, albeit weaker, was also observed for MtCHIT5b (electronic supplementary material, figure S6).

### 3.2. Enzyme activity of chimeras

**Figure 3.**
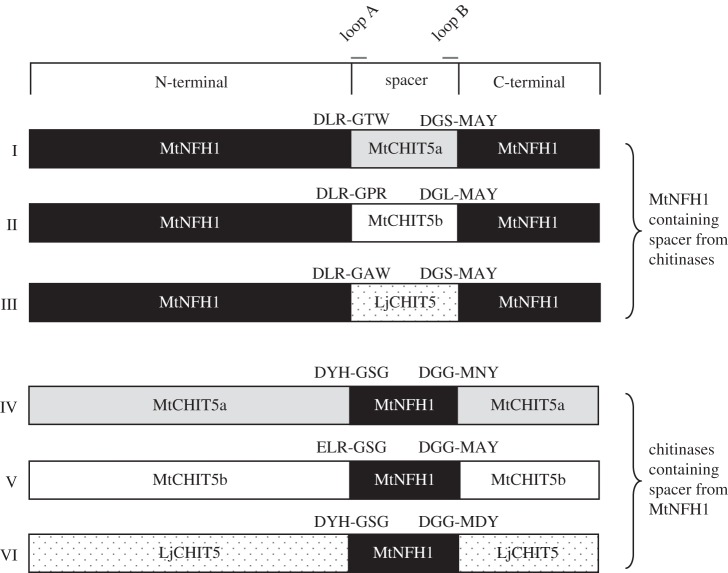
Schematic view of chimeras with hydrolytic activity. Three chimeras (constructs I–III) consist of MtNFH1 in which the spacer region (sequence from loop A to loop B) was replaced by the corresponding sequence of a chitinase (MtCHIT5a, MtCHIT5b and LjCHIT5). The three other constructs (constructs IV–VI) represent the chitinases containing the spacer region of MtNFH1.

Previous work suggests that the capacity of MtNFH1 to cleave NFs from *S. meliloti* is related to a distinct fatty acid-binding cleft. Two loops in MtNFH1 (loops A and B; figure 1) are predicted to interact with the fatty acid moiety [38]. To substantiate the role of a fatty acid-binding cleft for NF cleavage, we examined the enzyme activities of various chimeric proteins in which the sequence encompassing the residues from loop A to loop B was replaced by that of a homologue. Figure 3 shows a schematic view of these chimeras with their swapped sequence (named spacer region in this study; 74 amino acid residues from loop A to loop B; figure 1). MtNFH1 variants did not cleave *S. meliloti* NFs when the spacer region of MtNFH1 was replaced by that of the chitinases MtCHIT5a, MtCHIT5b or LjCHIT5 (chimeras I, II and III in figure 3). Instead, (GlcNAc)_6_, colloidal chitin, glycol chitin and CM–chitin–RBV were hydrolysed by these chimeras. Conversely, the chitinases MtCHIT5a, MtCHIT5b and LjCHIT5 containing the spacer region from MtNFH1 (chimeras IV, V and VI in figure 3) showed NF-cleaving activity, whereas the chitinous substrates were not degraded (table 2). These findings indicate that the substrate specificity of the examined chimeras depends on the spacer sequence and that the spacer sequences from the three legume chitinases were exchangeable in these chimeras. However, corresponding chimeras with sequences of MtNFH1 and the tobacco enzyme NtChiV lack hydrolytic activity (reciprocal chimeras VII and VIII; electronic supplementary material, figure S7).

**Table 2.** Activity of chimeric proteins.

substrate	enzyme activity (nkat mg^−1^)^a^					
	chimera I^b^	chimera II^b^	chimera III^b^	chimera IV^b^	chimera V^b^	chimera VI^b^
NodSm-V(C16 : 2, S)	ND^c^	ND	ND	129.9 ± 5.0	129.7 ± 6.8	126.4 ± 8.6
NodSm-IV(C16 : 2, S)	ND	ND	ND	116.6 ± 3.3	114.0 ± 9.4	115.8 ± 3.4
NodSm-IV(C16 : 2, Ac, S)	ND	ND	ND	34.0 ± 0.2	33.6 ± 1.1	33.3 ± 1.1
(GlcNAc)_6_	146.1 ± 19.3	168.6 ± 18.3	161.4 ± 5.8	ND	ND	ND
colloidal chitin	0.020 ± 0.006	0.020 ± 0.015	0.011 ± 0.006	ND	ND	ND
glycol chitin	11.3 ± 1.7	4.9 ± 0.7	5.4 ± 0.4	ND	ND	ND
CM–chitin–RBV	0.62 ± 0.23^d^	1.34 ± 0.04	1.36 ± 0.12	ND	ND	ND

^a^Enzyme assays were performed at 37°C with a substrate concentration of 150 µM for NodSm-V(C16 : 2, S), NodSm-IV(C16 : 2, S) and NodSm-IV(C16 : 2, Ac, S), 3.6 mM for (GlcNAc)_6_, approximately 10 mg ml^−1^ for colloidal chitin, 20 mg ml^−1^ for glycolchitin and 0.9 mg ml^−1^ for CM–chitin–RBV. Data indicate means ± s.e. from at least three independently purified enzyme preparations.

^b^Constructs I–III consist of MtNFH1 in which the spacer region (sequence from loop A to loop B) was replaced by the corresponding sequence of a chitinase (MtCHIT5a, MtCHIT5b and LjCHIT5). Constructs IV–VI represent chitinases containing the spacer region of MtNFH1 (figure 3).

^c^ND, not degraded.

^d^Enzyme activity expressed as ΔA_550_ **^.^** (mg protein)^−1^ s^−1^.

### 3.3. Activity of enzyme variants with modified loop A and loop B residues

The loops A and B (terminal ends of the spacer region) of MtCHIT5b and MtNFH1 differ in only four residues (figure 1). We therefore explored the enzymatic properties of MtNFH1 and MtCHIT5b variants in which loop A or loop B were modified. Deletion constructs were made to test the activity of MtNFH1 lacking loop A (ΔGSGS) or loop B (ΔPGPGVDGG). Both proteins failed to cleave *S. meliloti* NFs, providing support for the importance of these loops for NF hydrolysis (table 3). An MtCHIT5b variant, carrying the loop A and B sequences of MtNFH1 (P192S, R193G, S257P and L264G substitutions; figure 1), was able to cleave *S. meliloti* NFs. Moreover, MtCHIT5b(P192S R193G), i.e. MtCHIT5b with loop A from MtNFH1, showed NF-cleaving activity. Likewise, MtCHIT5b(S257P L264G), i.e. MtCHIT5b with the loop B sequence from MtNFH1, could cleave the NFs. Hence, swapping of two residues in either loop A or loop B of MtCHIT5b was sufficient to gain NF-cleaving activity. The MtCHIT5b variants retained the capacity to hydrolyse chitinous substrates, indicating bifunctional enzymes with chitinase and NF hydrolase activity (table 3).

**Table 3.** Activity of MtNFH1 and MtCHIT5b variants with modifications in loop A and loop B.

substrate	enzyme activity (nkat mg^−1^)^a^				
	MtNFH1 (ΔGSGS)^b^	MtNFH1 (ΔPGPGVDGG)^c^	MtCHIT5b(P192S, R193G, S257P, L264G)^d^	MtCHIT5b(P192S and R193G)^e^	MtCHIT5b(S257P and L264G)^f^
NodSm-V(C16 : 2, S)	ND^g^	ND	131.3±2.7	134.6±1.8	132.9±4.1
NodSm-IV(C16 : 2, S)	ND	ND	113.4±7.6	115.8±4.2	114.0±5.8
NodSm-IV(C16 : 2, Ac, S)	ND	ND	34.3±1.4	34.2±0.2	33.7±0.6
(GlcNAc)_6_	ND	ND	157.8±8.7	169.1±19.2	160.7±9.8
colloidal chitin	ND	ND	0.020±0.015	0.036±0.024	0.017±0.006
glycol chitin	ND	ND	11.7±0.8	10.7±0.9	8.1±0.5
CM–chitin–RBV	ND	ND	0.92±0.25^h^	0.42±0.04	0.62±0.05

^a^Enzyme assays were performed at 37°C with a substrate concentration of 150 µM for NodSm-V(C16 : 2, S), NodSm-IV(C16 : 2, S) and NodSm-IV(C16 : 2, Ac, S), 3.6 mM for (GlcNAc)_6_, approximately 10 mg ml^−1^ for colloidal chitin and 20 mg ml^−1^ for glycolchitin, and 0.9 mg ml^−1^ for CM–chitin–RBV. Data indicate means±s.e. from at least three independently purified enzyme preparations.

^b^Deletion of loop A.

^c^Deletion of loop B.

^d^MtCHIT5b variant with loops A and B of MtNFH1.

^e^MtCHIT5b variant with loop A of MtNFH1.

^f^MtCHIT5b variant with loop B of MtNFH1.

^g^ND, not degraded.

^h^Enzyme activity expressed as ΔA_550_ **^.^** (mg protein)^−1^ s^−1^.

Next, we systematically examined MtCHIT5b variants with single amino acid substitutions in the loops A or B. Proteins with a P192S, R193G or L264G substitution did not gain NF-cleaving activity. However, MtCHIT5b(S257P) (i.e. MtCHIT5b with the S257P substitution in loop B) showed NF hydrolysis. This finding indicates that a single serine-to-proline substitution in MtCHIT5b was sufficient to convert MtCHIT5b into an NF-cleaving enzyme. To further characterize the importance of this proline residue for NF hydrolysis, we replaced S257 of MtCHIT5b by alanine or lysine (corresponding residues in the loop B of the chitinases AtChiC of *A. thaliana* and NtChiV of tobacco, respectively; figure 1). Both proteins variants failed to cleave the *S. meliloti* NFs (table 4).

**Table 4.** Activity of MtCHIT5b variants with single amino acid substitutions in loop A or B using *S. meliloti* NFs and (GlcNAc)_6_ as substrates.

protein	enzyme activity (nkat mg^−1^)^a^		
	NodSm-V(C16 : 2, S)	NodSm-IV(C16 : 2, S)	(GlcNAc)_6_
MtCHIT5b	ND^b^	ND	134.4 ± 10.3
MtCHIT5b(P192S)^c^	ND	ND	128.6 ± 4.3
MtCHIT5b(R193G)^c^	ND	ND	120.1 ± 5.6
MtCHIT5b(S257P)^d^	127.2 ± 7.8^e^	116.6 ± 4.0	118.6 ± 4.9
MtCHIT5b(S257A)^d^	ND	ND	122.3 ± 7.8
MtCHIT5b(S257K)^d^	ND	ND	108.4 ± 6.9
MtCHIT5b(L264G)^d^	ND	ND	109.4 ± 12.3

^a^Enzyme assays were performed with indicated *S. meliloti* NFs (150 µM) and (GlcNAc)_6_ (4.5 mM).

^b^ND, not degraded.

^c^Substitution in loop A.

^d^Substitution in loop B.

^e^Mean values±s.e. from three independently purified enzyme preparations.

MtCHIT5b(S257P) showed NF-cleaving activities comparable to those of MtNFH1. MtCHIT5b(S257P) released the lipo-disaccharide NodSm-II(C16 : 2) from pentameric NodSm-V(C16 : 2, S) or tetrameric NodSm-IV(C16 : 2, S). Like MtNFH1, MtCHIT5b(S257P) also efficiently degraded NodSm-IV(C16 : 2, Ac, S) to the *O*-acetylated lipo-disaccharide NodSm-II(C16 : 2, Ac). Corresponding HPLC chromatograms (electronic supplementary material, figure S8) and mass spectrometry analysis data for the released acylated cleavage products (electronic supplementary material, table S3 and figure S9) provide detailed information. The Michaelis–Menten constants of MtCHIT5b(S257P) for the three *S. meliloti* NFs are in the micromolar range and nearly identical to those reported for MtNFH1 [38]. The *k*_cat_ values were also found to be similar to those determined for MtNFH1 [38] (table 5). Like MtCHIT5b (table 1), MtCHIT5b(S257P) can hydrolyse glycol chitin and CM–chitin–RBV (electronic supplementary material, table S4), whereas MtNFH1 is unable to degrade these substrates [38].

**Table 5.** *K*_m_ and *k*_cat_ values of MtCHIT5b(S257P) for *S. meliloti* NFs.

substrate	*K*_m_ (μM)	*k*_cat_ (s^−1^)	*k*_cat_ *K*_m_^−1^ (mM^−1^ s^−1^)
NodSm-V(C16 : 2, S)	49.8 ± 8.6^a^	7.9 ± 0.6	160.8 ± 15.3
NodSm-IV(C16 : 2, S)	58.6 ± 11.4	7.1 ± 0.7	122.1 ± 12.5
NodSm-IV(C16 : 2, Ac, S)	96.9 ± 18.8	5.5 ± 0.7	56.9 ± 4.4

^a^Means ± s.d. of *K*_m_ and *k*_cat_ values (37°C) deduced from kinetic data by using three independently purified enzyme preparations.

As S257P in MtCHIT5b is a gain-of-function substitution, we wondered whether MtNFH1 with a corresponding reverse substitution has an opposite effect. MtNFH1 with the serine-to-proline substitution P260S (first residue in loop B) completely lacked activity when *S. meliloti* NFs were used in the hydrolysis assay. Hence, MtNFH1(P260S) was found to be a loss-of-function variant. Like MtNFH1, MtNFH1(P260S) does not cleave (GlcNAc)_6_ (electronic supplementary material, table S5).

### 3.4. Homology modelling

**Figure 4.**
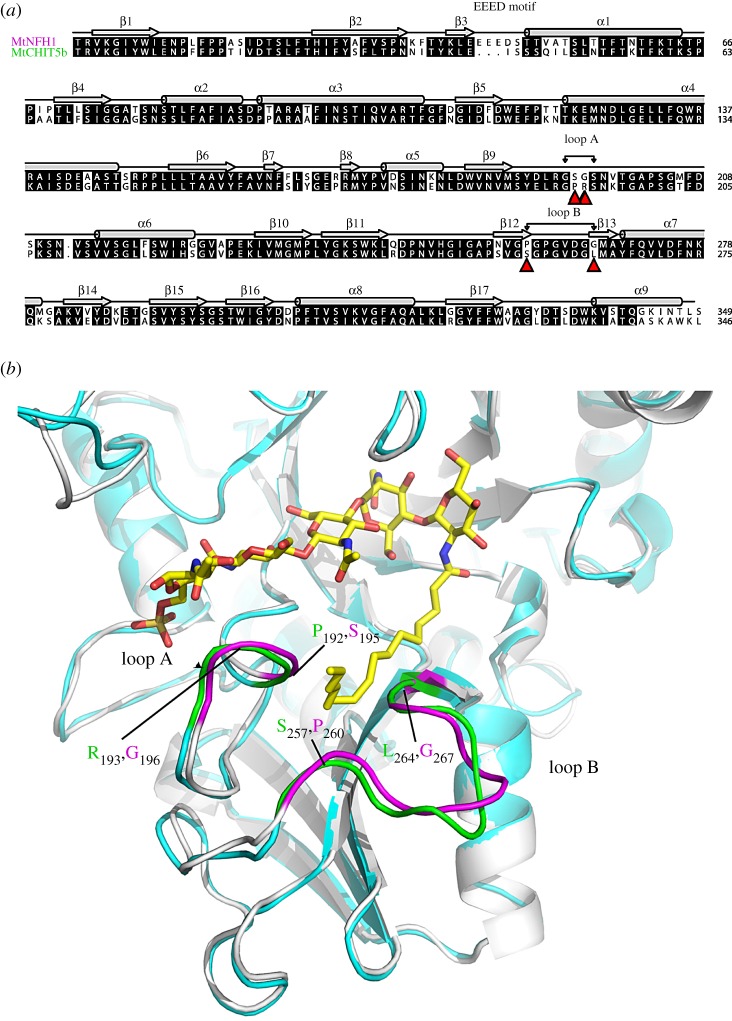
The three-dimensional models of MtNFH1 and MtCHIT5b are similar. (*a*) Sequence alignment of MtNFH1 (top) with MtCHIT5b (bottom). Identical amino acid residues are shown on a black background, and dashes indicate gaps. α-helices and β-strands are shown above the sequences. Different amino acid residues in the loops A and B are marked by red triangles. The EEED motif is only present in MtNFH1. Numbers of protein residues are marked on the right and correspond to the residues of the protein models. (*b*) Superimposition of models of MtNFH1 (grey) and MtCHIT5b (cyan) with NodSm-V(C16 : 2, S). The proteins are predicted to have a classic (β/α)_8_ triosephosphate isomerase (TIM) barrel fold with an additional (α + β) insertion domain (residues from β11 to β16). Loop A is part of the TIM-barrel domain and loop B is in the insertion domain. The NF was modelled in the binding pocket of MtNFH1 as reported previously [38]. The loops A and B of MtNFH1 (highlighted in magenta) are predicted to contribute to the formation of a cleft that accommodates the C16 : 2 fatty acid moiety of the NF substrate. Corresponding loops of MtCHIT5b are coloured green. Positions of different amino acid residues in the loops A and B are also marked.

Using the crystal structures of class V chitinases of *A. thaliana* (AtChiC) and tobacco (NtChiV) as structural templates, a three-dimensional model of MtNFH1 was constructed by homology modelling in our previous work [38]. Here, MtCHIT5b was modelled in a similar way. The structure-based alignment of the primary sequences used for homology modelling is shown in electronic supplementary material, figure S10 and an alignment of MtNFH1 with MtCHIT5b in figure 4*a*. Docking simulation of the NF to the substrate pocket of MtNFH1 was performed as reported previously [38] (figure 4*b*). As for MtNFH1, the loops A and B of MtCHIT5b are predicted to contribute to the formation of a cleft. The cleft in MtCHIT5b appears to be non-functional, however. Substitution of specific residues in loop A and B (P192S and R193G in loop A; S257P in loop B) results in MtCHIT5b variants with a modified cleft that can correctly accommodate the fatty acid chain of *S. meliloti* NFs. In other words, the gain-of-function properties of MtCHIT5b(P192S R193G) and MtCHIT5b(S257P) are probably associated with subtle structural changes required for proper binding of the C16 : 2 fatty acid moiety of *S. meliloti* NFs. However, these structural variations of loops A and B cannot be modelled accurately owing to poor alignment with the structural templates (electronic supplementary material, figure S10).

Furthermore, the performed docking simulation illustrates that the unique EEED motif in MtNFH1, which is absent in MtCHIT5b and other class V chitinase sequences (figure 1), is far away from the loops A and B. Hence, this MtNFH1-specific motif probably does not affect the physical interaction between MtNFH1 and the NF substrates (electronic supplementary material, figure S11).

## 4. Discussion

In this work, we have examined the MtNFH1-related proteins of *M. truncatula* and a closely related homologue of *L. japonicus* for their capacity to cleave chitinous substrates and NFs. In contrast to MtNFH1, none of the examined proteins was able to cleave NFs from *S. meliloti*. Instead, MtCHIT5a, MtCHIT5b and LjCHIT5 hydrolysed glycol chitin, CM–chitin–RBV, colloidal chitin and non-modified oligo-GlcNAc. Hence, these enzymes possess characteristics of classic class V chitinases as reported for tobacco [10–14], *A. thaliana* [15] and the cycad *C. revoluta* [17–19]. Accordingly, the Michaelis–Menten constants of MtCHIT5b for (GlcNAc)_6_ and (GlcNAc)_5_ were similar to those of NtChiV and AtChiC [38]. Chitinase activity of MtCHIT5a, MtCHIT5b and LjCHIT5 probably reflects a defence role against pathogenic fungi and other chitin-containing organisms. In fact, these enzymes inhibited hyphal growth of the fungus *T. viride* (electronic supplementary material, figure S6). This is reminiscent of the antifungal activity of the tobacco enzyme NtChiV [52]. Furthermore, available expression data (electronic supplementary material, table S1) and our additional qRT-PCR analysis for *MtCHIT5b* and *LjCHIT5* (electronic supplementary material, figure S3) indicate that transcripts of the examined class V chitinase genes are accumulated in response to pathogen attack. These findings suggest a function of these chitinases in plant defence. In contrast, recombinant MtCRA1 and MtCRA2 did not show any enzyme activity (figure 2), suggesting that these proteins represent lectins rather than enzymes. The recently published crystal structure of the homologue RobpsCRA of *R. pseudoacacia* provides explanations for the lack of enzyme activity [22]. RobpsCRA shows binding affinity to high-mannose-type N-glycans [21]. It remains to be elucidated whether MtCRA1 and MtCRA2 possess similar glycan-binding properties. The related Medtr3g110280 protein (not characterized in this study; see phylogenetic tree in electronic supplementary material, figure S1) is probably a third chitinase-like lectin of *M. truncatula*.

Neither MtNFH1 nor MtNFH1-related chitinases could cleave NFs from *R. leguminosarum* bv. *viciae* carrying a C18 : 4 acyl chain (table 1). In contrast, MtNFH1 was found to efficiently hydrolyse *S. meliloti* NFs but also NodSm-IV(C16 : 2), a chemically desulfated derivative [38]. Hence, the capacity of MtNFH1 to cleave *S. meliloti* NFs is not influenced by the reducing end modification of the sugar backbone (sulfate group) but rather depends on the acyl moiety. NFs carrying a C18 : 4 acyl chain obviously do not fit to the fatty acid cleft of MtNFH1 (figure 4*b*), indicating a high degree of substrate specificity. It is worth noting in this context that NFs of *R. leguminosarum* bv. *viciae* are rapidly degraded in the rhizosphere of the host plant *Vicia sativa* [40], whereas *S. meliloti* NFs are inactivated by MtNFH1 in the rhizosphere of the host *M. truncatula* [38]. The substrate preference of MtNFH1 for NFs with a C16 : 2 acyl chain, together with its symbiosis-related expression [37,39,41], corroborates the hypothesis that specific enzymes of host plants evolved to inactivate excess amounts of active NFs [34].

Class V chitinase family proteins differ in their capacity to cleave NFs and chitinous substrates. Previous work suggests that MtNFH1 is able to hydrolyse *S. meliloti* NFs, because the loops A and B of this enzyme contribute to a special protein structure that allows binding of the C16 : 2 acyl moiety [38]. This model is in agreement with the enzyme properties of reciprocal chimeras (figure 3 and table 2) and loss-of-function variants of MtNFH1 such as MtNFH1(P260S) (electronic supplementary material, table S5). Moreover, gain-of-function variants of MtCHIT5b, namely MtCHIT5b(P192S R193G) and MtCHIT5b(S257P) (tables 3 and 4), reinforce the requirement of a fatty acid-binding cleft in these proteins. It is intriguing that MtCHIT5b(S257P) and MtNFH1 [38] showed nearly identical enzyme kinetics when different *S. meliloti* NFs were used as substrates (table 5). Hence, the different substrate specificities of the NF hydrolase MtNFH1 and the chitinase MtCHIT5b can be attributed to distinct residues of the predicted fatty acid-binding cleft. The protein models obtained by homology modelling (figure 4) illustrate that the overall structures of MtCHIT5b and MtNFH1 are very similar. As for MtNFH1 [38], amino acid residues of the loops A and B of the engineered NF-cleaving MtCHIT5b variants probably interact with the C16 : 2 acyl chain of the NF substrate. Regarding MtCHIT5b with the gain-of-function substitution S257P and MtNFH1 with the corresponding loss-of-function substitution P260S, it can be assumed that the first proline residue in loop B (PGP motif in MtNFH1) creates a hydrophobic interaction surface that allows binding of the C16 : 2 acyl chain. Furthermore, the two-proline residue of the PGP motif may fix the backbone conformation of loop B and leave a cleft of sufficient width between the loops A and B. MtCHIT5a and LjCHIT5 have corresponding PAT and PAV motifs in loop B, suggesting that these chitinases (lacking NF hydrolase activity) are structurally different in this region. The lack of NF-cleaving activity in MtCHIT5b when compared with MtCHIT5b(P192S R193G) could be explained by steric hindrance effects. However, it should be noted that the accuracy of the performed homology modelling is limited and that we were unable to model subtle changes in the highly variable loops A and B.

The engineered NF-cleaving variants of MtCHIT5b with modifications in loop A and B are bifunctional enzymes that efficiently hydrolyse NFs and chitinous substrates (tables 3 and 4). In contrast, MtNFH1 has a specific substrate preference for NFs [38] (table 1); however, the protein models shown in figure 4 do not provide a structural explanation for this difference. It is worth noting, in this context, that MtNFH1 with a spacer region from MtCHIT5a, MtCHIT5b or LjCHIT5 could hydrolyse the chitinous substrates (chimeras I, II and III; table 2). These data indicate that the spacer region of these chitinases contains crucial residues that confer chitinase activity. On the other hand, MtNFH1 with the spacer region of the structurally different tobacco chitinase NtChiV did not show enzyme activity (chimera VII; electronic supplementary material, figure S7).

MtNFH1 contains a specific EEED motif that is absent in MtCHIT5b. This motif is due to a trinucleotide repeat expansion (microsatellite (AGA)_5_). The absence of this motif would considerably increase the isoelectric point of recombinant MtNFH1 (from 5.33 to 6.72). However, the EEED motif is far away from the fatty acid-binding cleft in our model (electronic supplementary material, figure S11), suggesting no influence on NF hydrolysis.

A systematic gain-of-function analysis to cleave *S. meliloti* NFs was performed for the chitinase MtCHIT5b. This enzyme is most similar to MtNFH1 and therefore was selected for further characterization in this study. The *MtCHIT5b* and *MtNFH1* genes are located in tandem on chromosome 4 of the *M. truncatula* genome (electronic supplementary material, figure S2), suggesting a gene duplication event during evolution. The EEED motif in MtNFH1 was probably generated after the gene duplication event. *MtCHIT5b* and *MtNFH1* can be considered as paralogues with different functions. We suggest that the capacity of MtNFH1 to cleave NFs is an evolutionary adaptation to the symbiosis with rhizobia. The evolution from a chitinase to an NF cleaving enzyme can be regarded as neofunctionalization [53]; that is, one gene copy (*MtCHIT5b)* retains the original defence-related chitinase function, whereas the second copy (*MtNFH1*) acquires the function to hydrolyse NFs. Gain-of-function mutations, similar to those experimentally performed in *MtCHIT5b*, probably also happened during evolution in ancestral forms of MtNFH1. Formation of a fatty acid-binding cleft for NFs was probably evolutionarily predisposed as MtCHIT5b appears to contain a similar (but non-functional) cleft in this region (figure 4*b*). Such a cleft is absent in the tobacco chitinase NtChiV that is unable to release lipo-disaccharides from *S. meliloti* NFs [38]. Slight modifications in the loops A or B of MtCHIT5b are apparently sufficient to convert the existing cleft into a functional fatty acid-binding cleft that allows bindings of NFs in a proper way. Likewise, ancestral forms of MtNFH1 were perhaps bi-functional chitinases/NF hydrolases during a transition stage and thus similar to MtCHIT5b(P192S R193G) and MtCHIT5b(S257P) characterized in this work. Additional mutations in *MtNFH1* (unknown residues in the spacer region) apparently caused loss of chitinase activity resulting in an enzyme with an entirely different substrate preference. Accordingly, chimeras IV, V, VI and VIII (chitinases carrying the spacer region of MtNFH1) do not cleave chitinous substrates (table 2; electronic supplementary material figure S7).

## 5. Concluding remarks

We found that MtCHIT5b can be converted into an NF-cleaving enzyme when a distinct serine residue in loop B is substituted by proline. Inversely, activity of MtNFH1 was abolished by a corresponding proline-to-serine substitution. These findings demonstrate that subtle changes in the shape of class V chitinases may have dramatic effects on cleavage of lipo-chitooligosaccharidic substrates. Evolution of an NF-cleaving enzyme from an ancestral chitinase can be considered as an example of symbiosis-related neofunctionalization. We currently perform tests with *M. truncatula* mutants to corroborate the symbiotic function of MtNFH1 during bacterial infection and within formed nodules. It is particularly intriguing that lipo-chitooligosaccharidic signal molecules are also produced by symbiotic mycorrhizal fungi (Myc factors) [54] and that certain host chitinases may have mycorrhiza-related functions [55]. Future studies on plant chitinases are needed to assess their ability to hydrolyse and inactivate Myc factors.

## Supplementary Material

Zhang et al SUPPLEMENTARY INFORMATION

## Data accessibility

Sequence data from this article can be found in the GenBank/EMBL data libraries under accession numbers KC833515 (MtNFH1), KC833513 (MtCHIT5a), KU041646 (MtCHIT5b), KC833514 (MtCRA1), KU041647 (MtCRA2) and KU041645 (LjCHIT5). Corresponding UniProt accession numbers are listed in electronic supplementary material, table S1.
