# Supplementary material for: A single amino acid substitution in a chitinase of the legume Medicago truncatula is sufficient to gain Nod-factor hydrolase activity
Source: Open Biol. 2016 Jul 6;6(7):160061. doi: 10.1098/rsob.160061 (PMC4967823; doi:10.1098/rsob.160061)
Supplement: Zhang et al SUPPLEMENTARY INFORMATION [file rsob160061supp1.pdf]

SUPPLEMENTARY INFORMATION

Title: A single amino acid substitution in a chitinase of the legume *Medicago truncatula* is sufficient to gain Nod-factor hydrolase activity.

Authors: Lan-Yue Zhang, Jie Cai, Ru-Jie Li, Wei Liu, Christian Wagner, Kam-Bo Wong, Zhi-Ping Xie & Christian Staehelin.

Supplementary Table S1. Overview of proteins characterized in this study.
Supplementary Table S2. Michaelis-Menten constants (K_m) and catalytic rate constants (k_{cat}) for MtCHIT5b with $(GlcNAc)_6$ and $(GlcNAc)_5$ as substrates.
Supplementary Table S3. MS analysis of purified NodSm-II(C16:2) and NodSm-II(C16:2, Ac) from reactions with *S. meliloti* NFs and MtCHIT5b(S257P).
Supplementary Table S4. MtCHIT5b(S257P) cleaves glycol chitin and CM-chitin-RBV.
Supplementary Table S5. MtNFH1(P260S) lacks hydrolytic activity.
Supplementary Table S6. Plasmids used in this study.
Supplementary Table S7. Primers used in this study.
Supplementary Figure S1. Unrooted phylogenic tree of MtNFH1 and proteins with related amino acid sequences.
Supplementary Figure S2. Genomic organization of *MtCHIT5b* and *MtNFH1* in chromosome 4 of *M. truncatula* ecotype Jemalong A17.
Supplementary Figure S3. Transcript levels of *MtCHIT5b* and *LjCHIT5* determined by qRT-PCR.
Supplementary Figure S4. Sodium dodecyl sulphate polyacrylamide gel electrophoresis and Western blot analysis of recombinant proteins.
Supplementary Figure S5. MALDI-TOF mass spectrometry analysis of degradation products released from $(GlcNAc)_6$ by MtCHIT5b.
Supplementary Figure S6. Fungal growth inhibition test with purified chitinases.
Supplementary Figure S7. Schematic view of chimeras lacking enzymatic activity.
Supplementary Figure S8. HPLC analysis of lipo-disaccharides released from *S. meliloti* NFs by MtCHIT5b(S257P).
Supplementary Figure S9. Positive-ion MALDI-TOF MS analysis of purified NodSm-II(C16:2) and NodSm-II(C16:2, Ac).
Supplementary Figure S10. Structure-based sequence alignment of MtNFH1 and MtCHIT5b.
Supplementary Figure S11. The EEEED motif of MtNFH1 is far away from the predicted fatty acid-binding cleft.

Supplementary Table S1. Overview of proteins characterized in this study.

Protein[a]	Accession number	Synonymous/ homologous	Amino acid identity with MtNFH1 (%)[b]	Elevated transcripts	Predicted signal peptide sequence[c]	Predicted *N*-glycosylation sites[d]	Molecular mass without signal peptide (kDa)[e]	pI[e]
MtNFH1	AJ515476[f] KC833515[g] U5N4E3[m]	Medtr4g116990.1 on chromosome 4[h]	100	Nodules, bumps and root hairs of *Medicago truncatula* plants inoculated with *Sinorhizobium meliloti* or treated with *S. meliloti* NFs[i, j,o]	MANFLKLKQ FLTLVLILLA LAAKSST	N114, N133, N232 and N246	39.0	5.3
MtCHIT5a	KC833513[g] U5N1E1[m]	Medtr1g013150.1 on chromosome 1[h]	49	Roots (inoculated with *Phymatotrichum* or treated with 200 mM NaCl); cells treated with yeast extract[j]	MAVQKIIITPI LVFLVTIFFN VSSSSS	N29, N114, N133, N195 and N234	40.3	8.8
MtCHIT5b	KU041646 A0A072UR65[m]	Medtr4g117000.1 on chromosome 4[h]	77	Plants inoculated with *Fusarium oxysporum*[k]	MANILNLKH LLTLALILLA LATKSS	N43, N84, N103, N167, N202 and N216	38.8	8.4
MtCRA2	KU041647 A0A072UR60[m]	Medtr4g116920.1 on chromosome 4[h]	34	Roots (treated with 200 mM NaCl)[j]	MAYSKKHSF LLISTLLIMIL QLQLFSTNA	N2, N77, N193 and N264	37.1	4.4
MtCRA1	KC833514[g] B7FNG8[m]	Medtr3g110320.1 on chromosome 3[h]	33	Seeds; cells treated with yeast extract[j]	MAYSKKHSF LLISTLLMIL QLQLS	N221, N233 and N304	37.0	8.5
LjCHIT5	KU041645 AK337657.1[n]	Lj0g3v0362579.1[h]	46	Nodules, particularly in-effective nodules[l]; plants inoculated with *F. oxysporum*[k]	MAVQSIITTP LLVILMSLRS YAFT	N112, N131, N193 and N231	39.8	7.4

[a]Amino acid sequences deduced from genomic DNA (*MtNFH1*, *MtCHIT5b* and *MtCRA2*) or cDNA (*MtCHIT5a* and *MtCRA1*) sequences of *Medicago truncatula* ecotype R108-1. LjCHIT5 deduced from cDNA of *Lotus japonicus* ecotype Miyakojima MG-20.
[b]Sequences without predicted signal peptide were compared.
[c]Signal peptides predicted by the SignalP program (http://www.cbs.dtu.dk/services/SignalP/).
[d]Predicted by the NetNGlyc 1.0 program (http://www.cbs.dtu.dk/services/NetNGlyc/).
[e]Theoretical values calculated with the Compute ProtParam tool (http://web.expasy.org/protparam/).
[f]Accession number reported by Salzer et al. 2004, *Planta*, 219, 628-638.
[g]Accession number reported by Tian et al. 2013, *Plant Physiol*, 163, 1179-1190.
[h]From the *M. truncatula* (ecotype Jemalong A17) genome version Mt4.0v1 (http://blast.jcvi.org/Medicago-Blast/index.cgi) and the *L. japonicus* genome database (http://www.kazusa.or.jp/lotus/index.html).
[i]Gene expression data of *M. truncatula* ecotype R108-1 as reported by Salzer et al. 2004, *Planta*, 219, 628-638.
[j]Gene expression data from the *M. truncatula* Gene Expression Atlas Web Server (http://mtgea.noble.org/v2).
[k]Expression of *MtCHIIT5b* and *LjCHIT5* analyzed by qRT-PCR (see Fig S3).
[l]Gene expression data from the *L. japonicus* Gene Expression Atlas Web Server (http://ljgea.noble.org/v2/).
[m]Accession number of the UniProt Consortium (The UniProt Consortium 2015, *Nucleic Acids Res* 43, D204-D212; http://www.uniprot.org/).
[n]Accession number of the NCBI database (http://www.ncbi.nlm.nih.gov/).
[o]*MtNFH1* expression data for root hairs of *M. truncatula* ecotype R108-1 as reported by Breakspear et al. 2014, *Plant Cell*, 26, 4680-4701.

Supplementary Table S2. Michaelis-Menten constants (K_m) and catalytic rate constants (k_{cat}) for MtCHIT5b with $(GlcNAc)_6$ and $(GlcNAc)_5$ as substrates.

Substrate	MtCHIT5b			MtNFH1[b]		
	K_m (mM)	k_{cat} (s^{-1})	$k_{cat}\,K_m^{-1}$ ($mM^{-1}\,s^{-1}$)	K_m (mM)	k_{cat} (s^{-1})	$k_{cat}\,K_m^{-1}$ ($mM^{-1}\,s^{-1}$)
$(GlcNAc)_6$	10.5[a]	78.6[a]	7.5	NA	NA	NA
$(GlcNAc)_5$	8.6	34.6	4.0	NA	NA	NA
NodSm-V(C16:2, S)	NA[c]	NA	NA	0.0527	8.4	159.3
NodSm-IV(C16:2, S)	NA	NA	NA	0.060	7.9	132.5
NodSm-IV(C16:2, Ac, S)	NA	NA	NA	0.0973	3.5	36.4

[a]K_m and k_{cat} values (37 °C) were deduced from HPLC chromatograms by integration of peak areas of formed cleavage products. Data were analyzed with the GraphPad Prism (version 5.00) software. Data indicate means from two independent experiments with similar results.

[b]Data for MtNFH1 reported by Tian et al. 2013 (*Plant Physiol*, 163, 1179-1190) are shown for comparison.

[c]NA, no activity.

Supplementary Table S3. MS analysis of purified NodSm-II(C16:2) and NodSm-II(C16:2, Ac) from reactions with *S. meliloti* NFs and MtCHIT5b(S257P).

Sample	Proposed structure	$[(M) +H]^+$		$[(M) +K]^+$	
		Predicted[a]	Observed	Predicted	Observed
NodSm-II(C16:2)[b]	NodSm-I(C16:2)[c]	396.54	396.16	NO[e]	NO
	NodSm-II(C16:2)	599.73	599.33	638.83	639.29
NodSm-II(C16:2, Ac)[d]	NodSm-I(C16:2, Ac)[c]	438.58	438.15	NO	NO
	NodSm-II(C16:2, Ac)	641.77	641.32	680.87	681.32

[a]Values were calculated from the average incremental masses of the component (without hydroxyl group at the reducing end).
[b]Released from NodSm-V(C16:2, S) by MtCHIT5b(S257P).
[c]Produced fragment ion.
[d]Released from NodSm-IV(C16:2, Ac, S) by MtCHIT5b(S257P).
[e]NO, not observed.

Supplementary Table S4. MtCHIT5b(S257P) cleaves glycol chitin and CM-chitin-RBV.

Substrate	Enzyme activity[a]
Glycolchitin	4.4 ± 0.5[b]
CM-chitin-RBV	0.09 ± 0.01[c]

[a]Enzyme assays with purified MtCHIT5b(S257P) were performed at 37°C with a substrate concentration of 20 mg mL^{-1} for glycolchitin and 0.9 mg mL^{-1} for CM-chitin-RBV. Data indicate means ± SD from at least three independently purified enzyme preparations. Values for MtCHIT5b without S257P substitution are shown in Table 1.

[b]Enzyme activity expressed as nkat · $(\text{mg protein})^{-1}$.

[c]Enzyme activity expressed as ΔA_{550} · $(\text{mg protein})^{-1}$ s^{-1}.

Supplementary Table S5. MtNFH1(P260S) lacks hydrolytic activity.

Substrate	Enzyme activity (nkat mg^{-1})[a]		Assay
	MtNFH1[b]	MtNFH1(P260S)	
NodSm-V(C16:2, S)	154.1 ± 28.2	ND	Reverse-phase HPLC analysis (C18 column)
NodSm-IV(C16:2, S)	116.7 ± 3.4	ND	
$(GlcNAc)_6$	ND	ND	Reverse-phase HPLC analysis (amino column)

[a]Enzyme assays were performed at 37°C with a substrate concentration of 150 µM for the NFs and 3.6 mM for $(GlcNAc)_6$. Data indicate means ± SD from at least three independently purified enzyme preparations. ND, no activity determined.

[b]Similar results were obtained previously (Tian et al., 2013, *Plant Physiol*, 163, 1179-1190).

Supplementary Table S6. Plasmids used in this study.

Plasmid	Relevant characteristics[a]	Used primers
pET28b	Expression vector based on pBR322 containing a 6xHis tag, Novagen/Merck, Darmstadt, Germany, Km^r.	
pET32a	Expression vector based on pBR322 containing 6xHis and Trx tags, Novagen/Merck, Darmstadt, Germany, Amp^r.	
pET-*MtNFH1*	pET28b derivative carrying a *NdeI-XhoI* fragment encoding MtNFH1 without predicted signal peptide, PCR amplified from genomic DNA of *M. truncatula* ecotype R108-1 (1.1 kb), Tian et al., 2013, *Plant Physiol*, 163, 1179-1190, Km^r.	1, 2
pET-*MtCHIT5a*	pET28b derivative carrying a *NdeI-XhoI* fragment encoding MtCHIT5a without predicted signal peptide, PCR amplified from cDNA of *M. truncatula* ecotype R108-1 (1.1 kb), Tian et al., 2013, *Plant Physiol*, 163, 1179-1190, Km^r.	3, 4
pET-*MtCHIT5b*	pET28b derivative carrying a *NdeI-XhoI* fragment encoding MtCHIT5b without predicted signal peptide, PCR amplified from genomic DNA of *M. truncatula* ecotype R108-1 (1.1 kb), Km^r.	5, 6
pET-*MtCRA1*	pET28b derivative carrying a *NdeI-XhoI* fragment encoding MtCRA1 without predicted signal peptide, PCR amplified from genomic DNA of *M. truncatula* ecotype R108-1 (1.1 kb), Tian et al., 2013, *Plant Physiol*, 163, 1179-1190, Km^r.	7, 8
pET-*MtCRA2*	pET32a derivative carrying a *NdeI-HindIII* fragment encoding MtCRA2 without predicted signal peptide, PCR amplified from genomic DNA of *M. truncatula* ecotype R108-1 (1.1 kb), Amp^r.	9a, 9b, 9c, 10a, 10b, 10c
pET-*LjCHIT5*	pET28b derivative carrying a *NdeI-XhoI* fragment encoding LjCHIT5 without predicted signal peptide (1.1 kb), PCR amplified from cDNA of *L. japonicus* ecotype MG-20, Km^r.	11, 12
pET-ChimeraI	pET-*MtNFH1* derivative encoding MtNFH1 with spacer region (from loop A to loop B) of MtCHIT5a, Km^r.	1, 2, 13, 14, 15, 16
pET-ChimeraII	pET-*MtNFH1* derivative encoding MtNFH1 with spacer region (from loop A to loop B) of MtCHIT5b, Km^r.	1, 2, 17, 18, 19, 20
pET-ChimeraIII	pET-*MtNFH1* derivative encoding MtNFH1 with spacer region (from loop A to loop B) of LjCHIT5, Km^r.	1, 2, 21, 22, 23, 24
pET-ChimeraIV	pET-*MtCHIT5a* derivative encoding MtCHIT5a with spacer region (from loop A to loop B) of MtNFH1, Km^r.	3, 4, 25, 26, 27, 28
pET-ChimeraV	pET-*MtCHIT5b* derivative encoding MtCHIT5b with spacer region (from loop A to loop B) of MtNFH1, Km^r.	5, 6, 29, 30, 31, 32
pET-ChimeraVI	pET-*LjCHIT5* derivative encoding LjCHIT5 with spacer	11, 12, 33, 34,

	region (from loop A to loop B) of MtNFH1, Km^{r}.	35, 36
pET-ChimeraVII	pET-*MtNFH1* derivative encoding MtNFH1 with spacer region (from loop A to loop B) of NtChiV (*Nicotiana tabacum*, PDB ID: 3ALF), Km^{r}.	1, 2, 73, 74, 75, 76
pET-ChimeraVIII	pET32a-*NtChiV* derivative encoding NtChiV with spacer region (from loop A to loop B) of MtNFH1, Amp^{r}.	77, 78, 79, 80, 81, 82
pET-*MtNFH1*(ΔGSGS)	pET-*MtNFH1* derivative encoding MtNFH1 lacking loop A, Km^{r}.	1, 2, 37, 38
pET-*MtNFH1*(ΔPGPGVDGG)	pET-*MtNFH1* derivative encoding MtNFH1 lacking loop B, Km^{r}.	1, 2, 39, 40
pET-*MtCHIT5b*(P192S, R193G)	pET-*MtCHIT5b* derivative encoding MtCHIT5b with P192S and R193G substitutions[b], Km^{r}.	41, 42
pET-*MtCHIT5b*(S257P)	pET-*MtCHIT5b* derivative encoding MtCHIT5b with a S257P substitution[b], Km^{r}.	43, 44
pET-*MtCHIT5b*(L264G）	pET-*MtCHIT5b* derivative encoding MtCHIT5b with a L264G substitution[b], Km^{r}.	45, 46
pET-*MtCHIT5b*(P192S)	pET-*MtCHIT5b* derivative encoding MtCHIT5b with a P192S substitution[b], Km^{r}.	47, 48
pET-MtCHIT5b(R193G)	pET-*MtCHIT5b* derivative encoding MtCHIT5b with a R193G substitution[b], Km^{r}.	49, 50
pET-*MtCHIT5b*(S257A)	pET-*MtCHIT5b* derivative encoding MtCHIT5b with a S257A substitution[b], Km^{r}.	51, 52
pET-*MtCHIT5b*(S257K)	pET-*MtCHIT5b* derivative encoding MtCHIT5b with a S257K substitution[b], Km^{r}.	53, 54
pET-*LjCHIT5*(D146A)	pET-*LjCHIT5* derivative encoding LjCHIT5 with a D146A substitution[c], Km^{r}.	55, 56
pET-*LjCHIT5*(W361A)	pET-*LjCHIT5* derivative encoding LjCHIT5 with a W361A substitution[c], Km^{r}.	57, 58
pET-*MtCHIT5a*(D148A)	pET-*MtCHIT5a* derivative encoding MtCHIT5a with a D148A substitution[c], Km^{r}.	59, 60
pET-*MtCHIT5a*(W364A)	pET-*MtCHIT5a* derivative encoding MtCHIT5a with a W364A substitution[c], Km^{r}.	61, 62
pET-*MtNFH1*(P260S)	pET-*MtNFH1* derivative encoding MtNFH1 with a P260S substitution[b], Km^{r}.	63, 64

[a]Km^{r}, Amp^{r} and Tc^{r}: Resistance to kanamycin, ampicillin and tetracycline, respectively.

[b]Numbering of residues based on the alignment shown in Fig. S10.

[c]Numbering of residues based on predicted protein sequences (accession numbers KC833513 and KU041645).

Supplementary Table S7. Primers used in this study.

Primer	Sequence (5’ to 3’)[a]	Restriction site	Description
1	CAAAAGTCATATGAGCACAACATCACCATCATCAAC	*Nde*I	Amplification of the coding region of *MtNFH1* without predicted signal peptide from genomic DNA of *M. truncatula* ecotype R108-1 and insertion into pET28b, generating pET-*MtNFH1.*
2	GCCTCGAGTTAAGAAAGTGTGTTAATTTTACC	*XhoI*	
3	GGAATTCCATATGTCTAATAATTCGCAATACCAATTTT	*NdeI*	Amplification of the coding region of *MtCHIT5a* without predicted signal peptide from cDNAs of *M. truncatula* ecotype R108-1 and insertion into pET28b, generating pET-*MtCHIT5a.*
4	CCGCTCGAGTCAGTGTCCCCATGCATTTGAAG	*XhoI*	
5	CAAAAGTCATATGTCATCATCATCATCAATCACACGTGT	*Nde*I	Amplification of the coding region of *MtCHIT5b* without predicted signal peptide from genomic DNA of *M. truncatula* ecotype R108-1 and insertion into pET28b, generating pET-*MtCHIT5b.*
6	GCCTCGAGTCACACAAGCTTCCAGGCTTTCGAAGCTTTCGAAGCTTGAGTTGCG	*Xho*I	
7	TACAAATCATATGATTAAAGGTGGCTATTGG	*Nde*I	Amplification of the coding region of *MtCRA1* without predicted signal peptide from genomic DNA of *M. truncatula* ecotype R108-1 and insertion into pET28b, generating pET-*MtCRA1.*
8	TACTCGAGCTAGGTCCCCCAGTTACGAGAAGC	*Xho*I	
9a	CTCTACAGGATCCCTCCATTAAAGGTGGCTATTGG	*BamH*I	Amplification of the coding region of *MtCRA2* without predicted signal peptide from genomic DNA of *M. truncatula* ecotype R108-1 and insertion into pET32a, generating pET-*MtCRA2.*
9b	GAAGTTGTTATTTTGGCTTGGCTAGCC		
9c	GGCTAGCCAAGCCAAAATAACAACTTC		

10a	CCAACAAATATATAGGCCTGCTT GAGAAAG		
10b	CTTTCTCAAGCAGGCCTATATAT TTGTTGG		
10c	CCCAAGCTTCTAGGTGAAGAGC AACTTCCTTAATC	*Hind*III	
11	GGGAATTCCATATGGAACCTTCT TTACATAGGCAGC	*Nde*I	Amplification of the coding region of *LjCHIT5* without predicted signal peptide from cDNAs of *L. japonicus* ecotype MG-20 and insertion into pET28b, generating pET-*LjCHIT5*.
12	CCGCTCGAGTCAGTATCCCCAT GCATTTGAAG	*Xho*I	
13	TTATTATCCCACGTGCCGTGGCG AAGATCATAACTCATAA		Domain swapping by overlapping extension PCR with pET-*MtNFH1* and pET-*MtCHIT5a* as template, generating pET-ChimeraI.
14	TTATGAGTTATGATCTTCGCCAC GGCACGTGGGATAATAA		
15	ACAACTTGAAAATACGCCATAC CATCCGTGT		
16	CTGCAACTGACACGGATGGTAT GGCGTATTTTCAAGTTGT		
17	GTTTTATTACTCCGTGGCCCGCG AAGATCATAACTCATAA		Domain swapping by overlapping extension PCR with pET-*MtNFH1* and pET-*MtCHIT5b* as template, generating pET-ChimeraII.
18	TTATGAGTTATGATCTTCGCGGG CCACGGAGTAATAAAAC		
19	ACAACTTGAAAATACGCCATTA AACCATCAACCCCAGGAC		
20	GTCCTGGGGTTGATGGTTTAAT GGCGTATTTTCAAGTTGT		
21	GTTTCGTTGGCCCACGCGCCGC		Domain swapping by overlapping extension PCR

	GAAGATCATAACTCATAAC	with pET-*MtNFH1* and pET-*LjCHIT5* as template, generating pET-ChimeraIII.
22	TATGAGTTATGATCTTCGCGGCG CGTGGGCCACG	
23	ATCCACAACTTGAAAATACGCC ATACTACCATCTGTATCAACTG	
24	GTTGATACAGATGGTAGTATGGC GTATTTTCA	
25	GTCACATTACTACCCGACCCATA ATCGAAACACATTGGAC	Domain swapping by overlapping extension PCR with pET-*MtNFH1* and pET-*MtCHIT5a* as template, generating pET-ChimeraIV.
26	GTCCAATGTGTTTCGATTATGGG TCGGGTAGTAATGTGAC	
27	ATCTCATTATAATTCATACTTCCA CCATCAACCCCAGGAC	
28	GTCCTGGGGTTGATGGTGGAAG TATGAATTATAATGAGAT	
29	GTCACATTACTACCCGACCCAC GAAGTTCATAACTCATAA	Domain swapping by overlapping extension PCR with pET-*MtNFH1* and pET-*MtCHIT5b* as template, generating pET-ChimeraV.
30	TTATGAGTTATGAACTTCGTGGG TCGGGTAGTAATGTGAC	
31	AACACTTGGAAATACGCCATTC CACCATCAACCCCAGGA	
32	GTCCTGGGGTTGATGGTGGAAT GGCGTATTTCCAAGTGTT	
33	CATTACTACCCGACCCATTGTAA TCAAAAC	Domain swapping by overlapping extension PCR with pET-*MtNFH1* and pET-*LjCHIT5* as template, generating pET-ChimeraVI.
34	GTTTTGATTACAATGGGTCGGGT AGTAATG	
35	GATTTCATCATAGTCCATTCCAC CATCAAC	

36	GTTGATGGTGGAATGGACTATG ATGAAATC	
37	ATGGAGCCCCGGTCACATTGCG AAGATCATAACTCATA	Deletion of loop A by overlapping extension PCR with pET-*MtNFH1* as template, generating pET-*MtNFH1*(ΔGSGS).
38	TATGAGTTATGATCTTCGCAATG TGACCGGGG	
39	CAACTTGAAAATACGCCATTCC AACATTCGGTGCCCCG	Deletion of loop B by overlapping extension PCR with pET-*MtNFH1* as template, generating pET-*MtNFH1*(ΔPGPGVDGG).
40	CGGGGCACCGAATGTTGGAATG GCGTATTTTCAAGTTG	
41	TTATGAACTTCGTGGGTCGGGT AGTAATAAAACCGGGG	Site-directed mutagenesis by overlapping extension PCR with pET-*MtCHIT5b* as template, generating pET-*MtCHIT5b*(P192S, R193G).
42	GCCCCGGTTTTATTACACCCGA CCCACGAAGTTCATA	
43	GGCACCGAGTGTTGGGCCGGG TCCTGGGGTTGATG	Site-directed mutagenesis by overlapping extension PCR with pET-MtCHIT5b as template, generating a S257P substitution in MtCHIT5b.
44	CATCAACCCCAGGACCCGGCCC AACACTCGGTGCC	
45	GGTCCTGGGGTTGATGGTGGAA TGGCGTATTTCCAAGTG	Site-directed mutagenesis by overlapping extension PCR with pET-MtCHIT5b as template, generating a L264G substitution in MtCHIT5b.
46	CACTTGGAAATACGCCATTCCA CCATCAACCCCAGGACC	
47	GAACTTCGTGGGTCGCGGAGTA ATAAA	Site-directed mutagenesis by overlapping extension PCR with pET-*MtCHIT5b* as template, generating pET-*MtCHIT5b*(P192S).
48	AAATAATGAGGCGCTGGGTGCT TCAAG	
49	CTTCGTGGGCCAGGTAGTAATA AAACC	Site-directed mutagenesis by overlapping extension PCR with pET-*MtCHIT5b* as template,

50	GGTTTTATTACTACCTGGCCCACGAAG	generating pET-*MtCHIT5b*(R193G).
51	GGCACCGAGTGTTGGGGCGGGTCCTGGGGTTGATG	Site-directed mutagenesis by overlapping extension PCR with pET-*MtCHIT5b* as template, generating a S257A substitution in MtCHIT5b.
52	CATCAACCCCAGGACCCGCCCCAACACTCGGTGCC	
53	GGCACCGAGTGTTGGGAAGGGTCCTGGGGTTGATG	Site-directed mutagenesis by overlapping extension PCR with pET-*MtCHIT5b* as template, generating a S257K substitution in MtCHIT5b.
54	CATCAACCCCAGGACCCTTCCCAACACTCGGTGCC	
55	GAGTTCAACGGTCTTGCCTTGGATTGGGAGTGG	Site-directed mutagenesis by overlapping extension PCR with pET-*LjCHIT5* as template, generating a D146A substitution in LjCHIT5.
56	CCACTCCCAATCCAAGGCAAGACCGTTGAA	
57	AAGGGGTATTTCTTTGCCGCTATTGGGAAGGAC	Site-directed mutagenesis by overlapping extension PCR with pET-*LjCHIT5* as template, generating a W361A substitution in LjCHIT5.
58	GTCCTTCCCAATAGCGGCAAAGAAATACCCCTT	
59	AGATTTGATGGTGTTGCCTTGGATTGGGAATTT	Site-directed mutagenesis by overlapping extension PCR with pET-*MtCHIT5a* as template, generating a D148A substitution in MtCHIT5a.
60	AAATTCCCAATCCAAGGCAACACCATCAAATCT	
61	AAGGGCTATTTCTTTGCCGCCCTTGGGAAGGAC	Site-directed mutagenesis by overlapping extension PCR with pET-*MtCHIT5a* as template, generating a W364A substitution in MtCHIT5a.
62	GTCCTTCCCAAGGGCGGCAAAGAAATAGCCCTT	
63	CCGAATGTTGGATCGGGTCCTGGGGTT	Site-directed mutagenesis by overlapping extension PCR with pET-*MtNFH1* as template, generating a P260S substitution in MtNFH1.
64	AACCCCAGGACCCGATCCAACA	

	TTCGG		
65	AGTGGAAGTACGTGGATCGGATACGATA		Amplification of a *MtCHIT5b* fragment; for qRT-PCR.
66	TCCAGGCTTTCGAAGCTTGAGTTGCGAT		
67	ATGTTGCTATTCAGGCCGTTCTTTC		Amplification of a *Mtactin-97* fragment (accession: XM_003621971); for qRT-PCR.
68	AGCCAGATCAAGACGAAGGATGG		
69	TTCACCTTGTGCTCCGTCTTC		Amplification of a *Ljubiquitin* fragment (accession: DQ24991); for qRT-PCR.
70	AACAACAGCACACACAGACAATC		
71	GTTGTGTATGATGAAGTGGCTGTG		Amplification of a *LjCHIT5* fragment; for qRT-PCR.
72	ATCCCCATGCATTTGAAGCTTGTT		
73	GAGTTATGATCTTCGCGGACCAAATTGGTC		Domain swapping by overlapping extension PCR with pET-*MtNFH1* and pET-*NtChiV* as template, generating pET-ChimeraVII.
74	GACCAATTTGGTCCGCGAAGATCATAACTC		
75	GATGATGGGTCGATGGCGTATTTTCAAG		
76	CTTGAAAATACGCCATCGACCCATCATC		
77	GGAATTCCAAAATGTTATTAAGGGAGGGTACTGG	*Eco*RI	Domain swapping by overlapping extension PCR with pET-*MtNFH1* and pET-*NtChiV* as template, generating pET32a-ChimeraVIII.
78	GGCATATGACTTCTATGGGTCGGGTAG		

79	CTACCCGACCCATAGAAGTCATA TGCC	
80	GATGGTGGAATGACTTATAACA G	
81	CTGTTATAAGTCATTCCACCATC	
82	GCCTCGAGTCACTTCATCTCTTG AGATGACACTCCC	*Xho*I

[a]Restriction sites in nucleotide sequences are underlined; substituted nucleotides are framed.

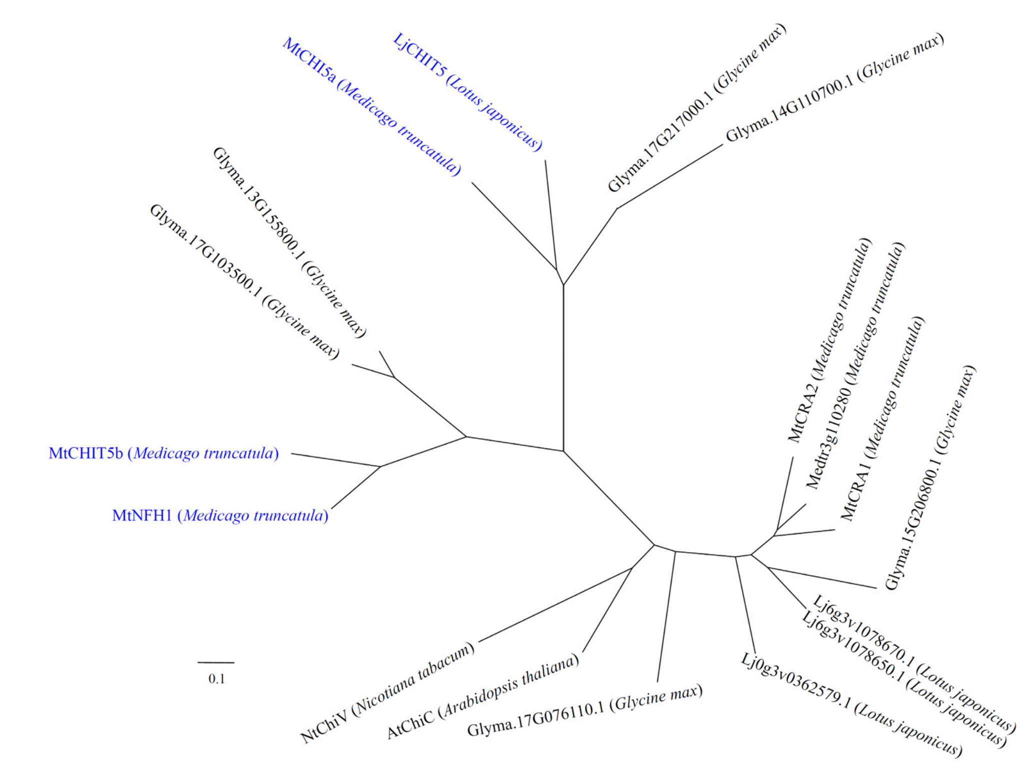

Supplementary Figure S1. Unrooted phylogenic tree of MtNFH1 and related amino acid sequences of *M. truncatula, L. japonicus* and *Glycine max*. The reference proteins AtChiC and NtChiV were also included. The tree was constructed with the MEGA5.0 program using the neighbor-joining method (Tamura et al. 2011, *Mol Biol Evol*, 28, 2731-2739). MtNFH1 and the three closely related chitinases characterized in this study (MtCHIT5a, MtCHIT5b and LjCHIT5) are highlighted in blue. The horizontal bar represents a distance of 0.1 substitutions per site. The sequences and accession numbers are as follows: (i) *M. truncatula* ecotype R108-1: MtNFH1 (KC833515), MtCHIT5a (KC833513.1), MtCHIT5b (KU041646), MtCRA1 (KC833514.1), MtCRA2 (KU041647) and Medtr3g110280 (XP_013462049.1); (ii) *L. japonicus* ecotype Miyakojima MG-20: LjCHIT5 (KU041645), Lj0g3v362579.1, Lj6g3v1078650.1 (LjCRA; Sulzenbacher et al. 2015, *J Struct Biol*, 190, 115-121; AFK36566.1), and Lj6g3v1078670.1 (AFK36566.1); (iii) reference sequences: AtChiC of *A. thaliana* (NP_193716, 3AQU) and NtChiV of *N. tabacum* (CAA55128, CAA54373, 3ALF). Further related sequences not shown in the phylogenic tree: (i) Sequences encoding putative receptor-like kinase proteins (Medtr4g117020.1, Medtr4g117030.1, Medtr4g117040.1, Lj1g3v0177810.1 and Glyma.15G206400.1) and (ii) incomplete sequences lacking N or C-terminal ends (such as Lj5g3v0525250.1, Lj5g3v0525260.1, Lj0g3v0284719.1, Lj5g3v0526340.1, Lj0g3v0284689.1 and Medtr4g117800.1).

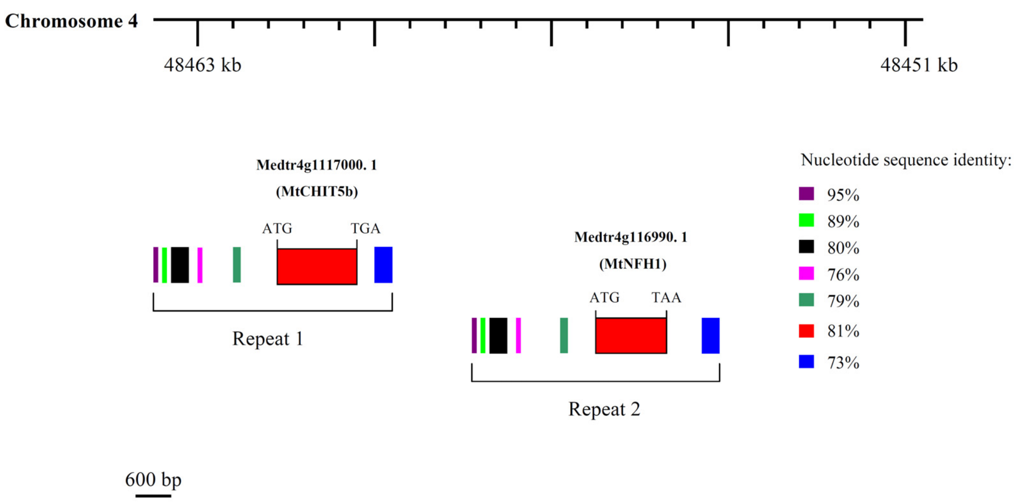

Supplementary Figure S2. Genomic organization of *MtCHIT5b* and *MtNFH1* in chromosome 4 of *M. truncatula* ecotype Jemalong A17. Medtr4g117000.1 (corresponds to *MtCHIT5b* of *M. truncatula* ecotype R108-1) and Medtr4g116990.1 (corresponds to *MtNFH1* of *M. truncatula* ecotype R108-1) are located in tandem at position 48461626-48460382 and 48456188-48454821, respectively. Same colours indicate regions with high nucleotide sequence identity (>70%).

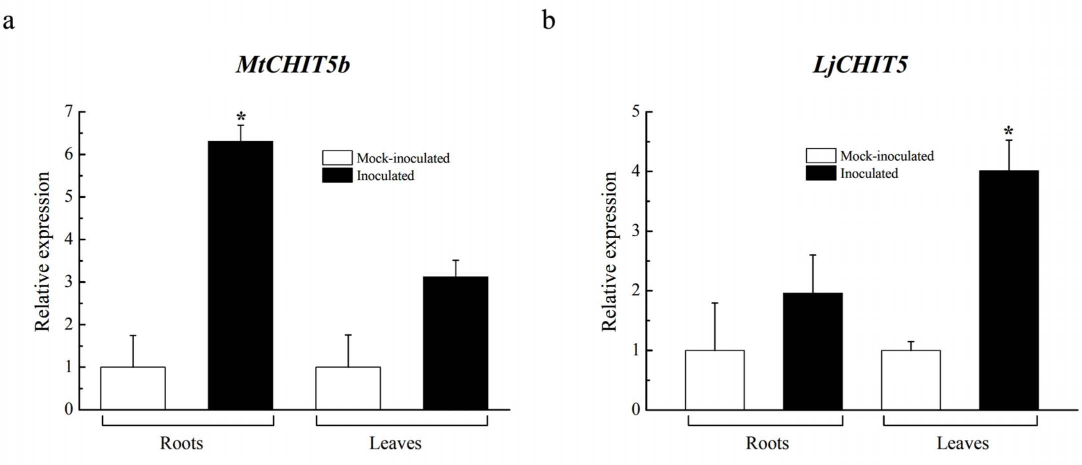

Supplementary Figure S3. Transcript levels of *MtCHIT5b* and *LjCHIT5* determined by qRT-PCR. *M. truncatula* (ecotype R108-1) and *L. japonicus* (ecotype Miyakojima MG-20) were inoculated with *Fusarium oxysporum* f. sp. cubense race 4 (1.6×10^6 spores ml^{-1}) or mock-inoculated (control). RNA was isolated from roots and leaves of plants harvested 20 (*M. truncatula*) and 26 (*L. japonicus*) days post inoculation. Data represent mean values (±SE) of three independent experiments with RNA extracted from three *M. truncatula* and *L. japonicus* plants (n = 3). All PCRs were performed in triplicate. The *Mtactin-97* gene of *M. truncatula* and the *Ljubiquitin* gene of *L. japonicus* were used as references. Asterisks mark significant differences between inoculated and mock-inoculated plants (Student's t test: $p<0.05$). (**a**) *MtCHIT5b*. (**b**) *LjCHIT5*.

a

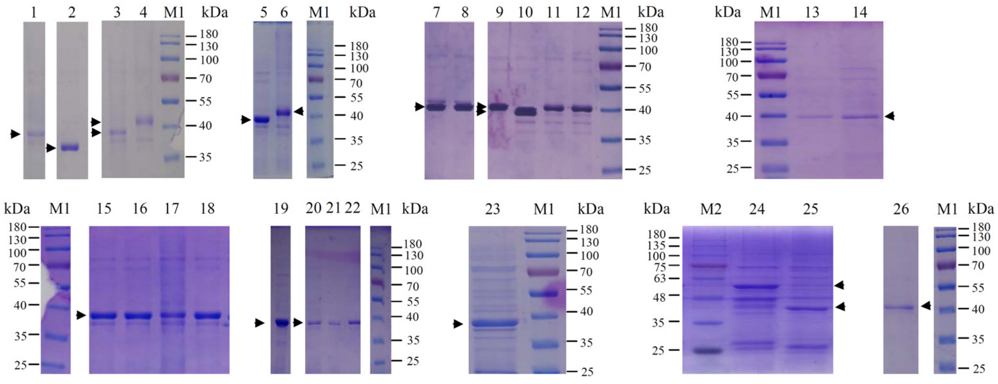

b

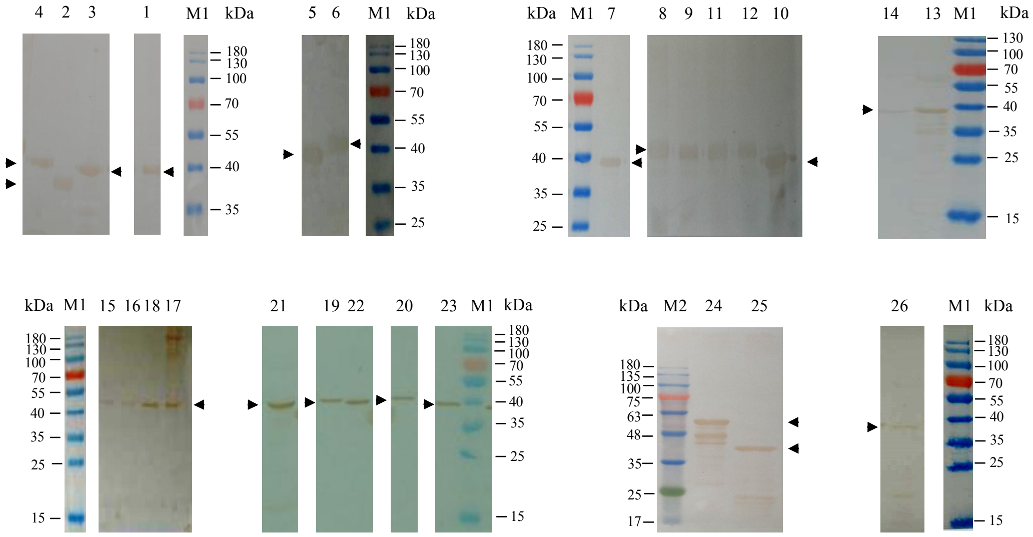

Supplementary Figure S4. Sodium dodecyl sulphate polyacrylamide gel electrophoresis (SDS-PAGE) and Western blot analysis of recombinant proteins. The 6xHis-tagged proteins were expressed in *E. coli* BL21 (DE3) and purified by nickel affinity chromatography. (**a**) SDS-PAGE analysis of recombinant proteins. Gels were stained with Coomassie Brilliant Blue R-250. (**b**) Western blot analysis of recombinant proteins. Blots were incubated with rabbit serum raised against MtNFH1 and developed with the 3,3′-diaminobenzidine reagent. Following proteins were analysed (arrowheads): 1: MtCHIT5a; 2: MtCRA1; 3: MtNFH1; 4: MtCHIT5b; 5: LjCHIT5; 6: MtCRA2 (with additional Trx tag); 7: Chimera II; 8: Chimera I; 9: Chimera IV; 10: Chimera V; 11: Chimera III; 12: Chimera VI; 13: MtNFH1(ΔGSGS); 14: MtNFH1(ΔPGPGVDGG); 15: MtCHIT5b(P192S, R193G, S257P, L264G); 16: MtCHIT5b(S257P and L264G); 17: MtCHIT5b(L264G); 18: MtCHIT5b(P192S and R193G); 19: MtCHIT5b(S257A); 20: MtCHIT5b(S257K); 21: MtCHIT5b(S257P); 22: MtCHIT5b(R193G); 23: MtCHIT5b(P192S); 24: Chimera VIII (with additional Trx tag); 25: Chimera VII; 26: MtNFH1(P260S); M1 and M2: Molecular weight markers 1 and 2, respectively. Adjustments of brightness and contrast were performed for Western blot images (proteins 5-18 and 24-26).

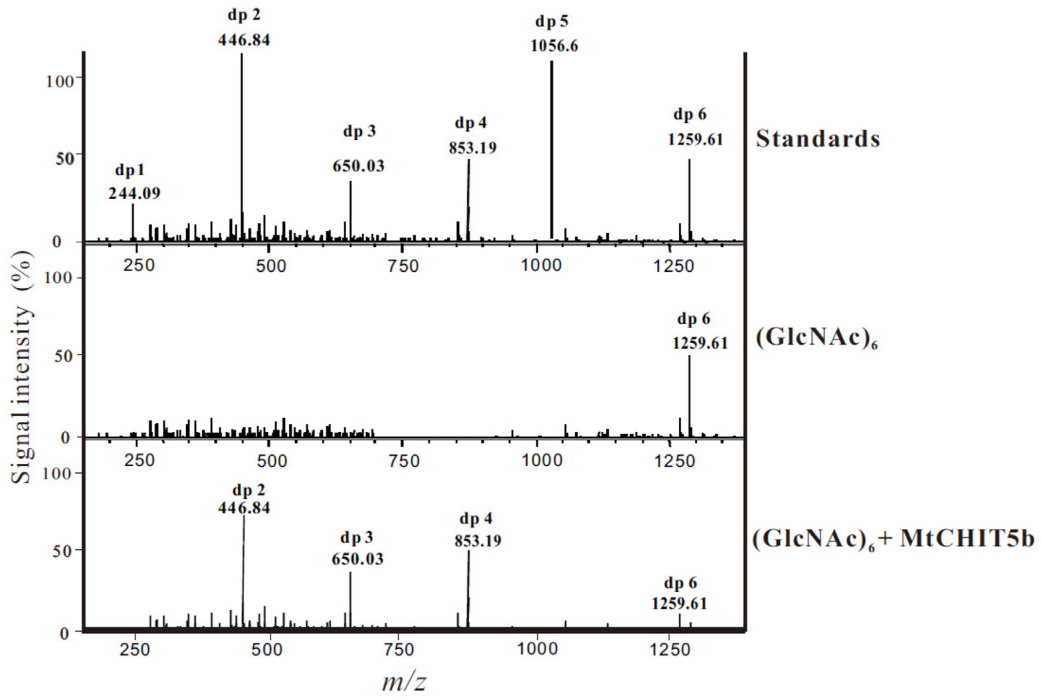

Supplementary Figure S5. MALDI-TOF mass spectrometry analysis of degradation products released from $(GlcNAc)_6$ by MtCHIT5b. Oligo-GlcNAc with indicated degree of polymerisation (dp) in 4 mM sodium acetate buffer (pH 5.0) were used as standards (top). $(GlcNAc)_6$ in the same buffer was used as substrate (middle). 5.8 mM $(GlcNAc)_6$ incubated with 5.0 µg ml^{-1} MtCHIT5b for 30 min in the buffer at 37 ºC (bottom). Numbers above signals indicate *m/z* values of fragment ions lacking the hydroxyl group at the reducing end (positive-ion mode).

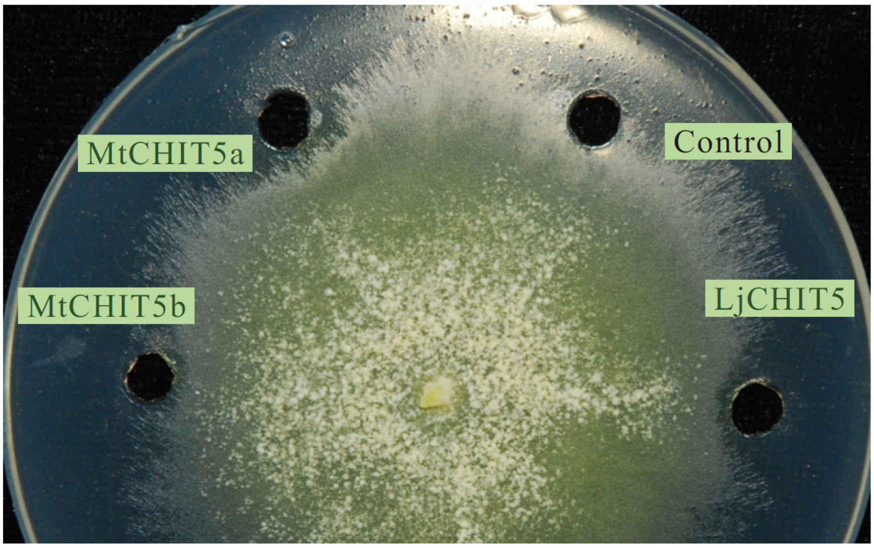

Supplementary Figure S6. Fungal growth inhibition test with purified chitinases. 3.2 µg of indicated enzyme in 50 µl of 25 mM sodium acetate buffer (pH 5.0) were pipetted into each well close to a growing mycelium of *Trichoderma viride*. The buffer without enzyme was used as a control. The plate was incubated at 27 °C for 14 h and then photographed.

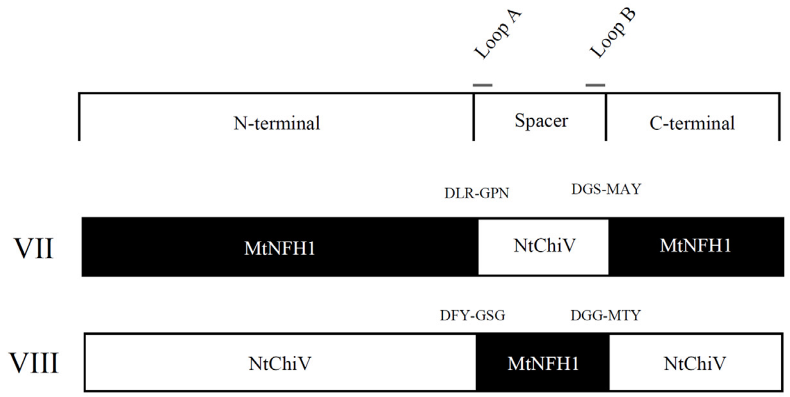

Supplementary Figure S7. Schematic view of chimeras lacking enzymatic activity. Chimera VII consists of MtNFH1 in which the spacer region (sequence from loop A to loop B) was replaced by the corresponding sequence of NtChiV (class 5 chitinase of *Nicotiana tabacum*; accession no. X78325). Chimera VIII represents NtChiV containing the spacer region of MtNFH1. Both chimeras lack hydrolytic activity as examined with *S. meliloti* NFs, $(GlcNAc)_6$, glycolchitin and CM-chitin-RBV.

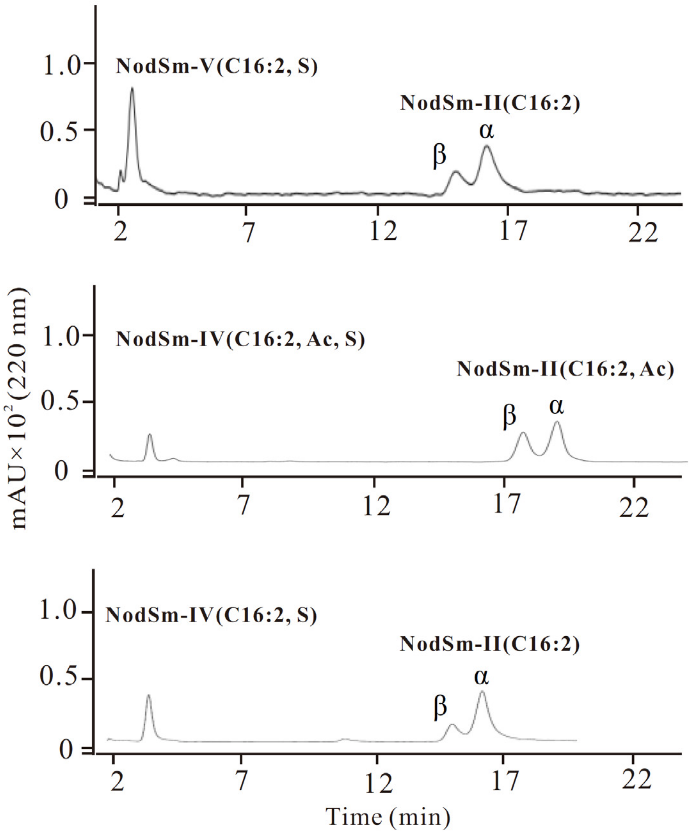

Supplementary Figure S8. HPLC analysis of lipo-disaccharides released from *S. meliloti* NFs by MtCHIT5b(S257P). Indicated NFs and formed lipo-disaccharides were separated on a Nova Pak C18 column. The lipo-disaccharides were separated into anomers (double peaks). 0.9 µg ml^{-1} MtCHIT5b(S257P) was incubated with 40 µM NodSm-V(C16:2, S) for 20 min (top), with 40 µM NodSm-IV(C16:2, Ac, S) for 20 min (middle), and with 60 µM NodSm-IV(C16:2, S) for 20 min (bottom).

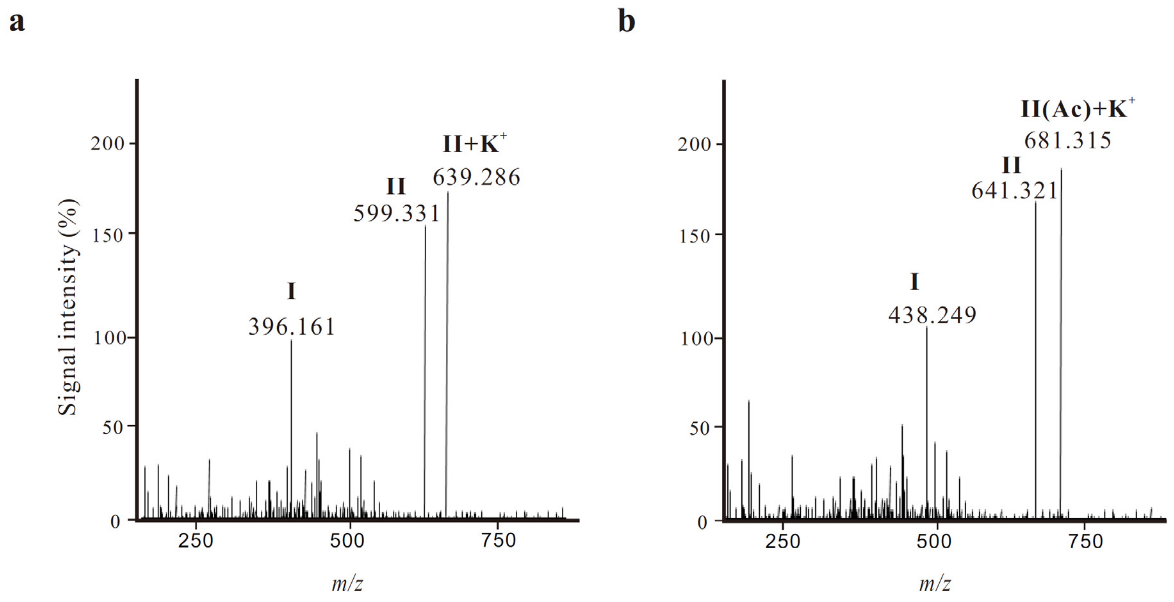

Supplementary Figure S9. Positive-ion MALDI-TOF MS analysis of purified NodSm-II(C16:2) and NodSm-II(C16:2, Ac). (**a**) NodSm-II(C16:2) released from NodSm-IV(C16:2, S) by MtCHIT5b(S257P). The signal I corresponds to the fragment ion NodSm-I(C16:2) and the signal II+K^+ is a potassium adduct. (**b**) NodSm-II(C16:2, Ac) released from NodSm-IV(C16:2, Ac, S) by MtCHIT5b(S257P). The signal I (Ac) corresponds to the fragment ion NodSm-I(C16:2, Ac) and II (Ac)+K^+ is a potassium adduct.

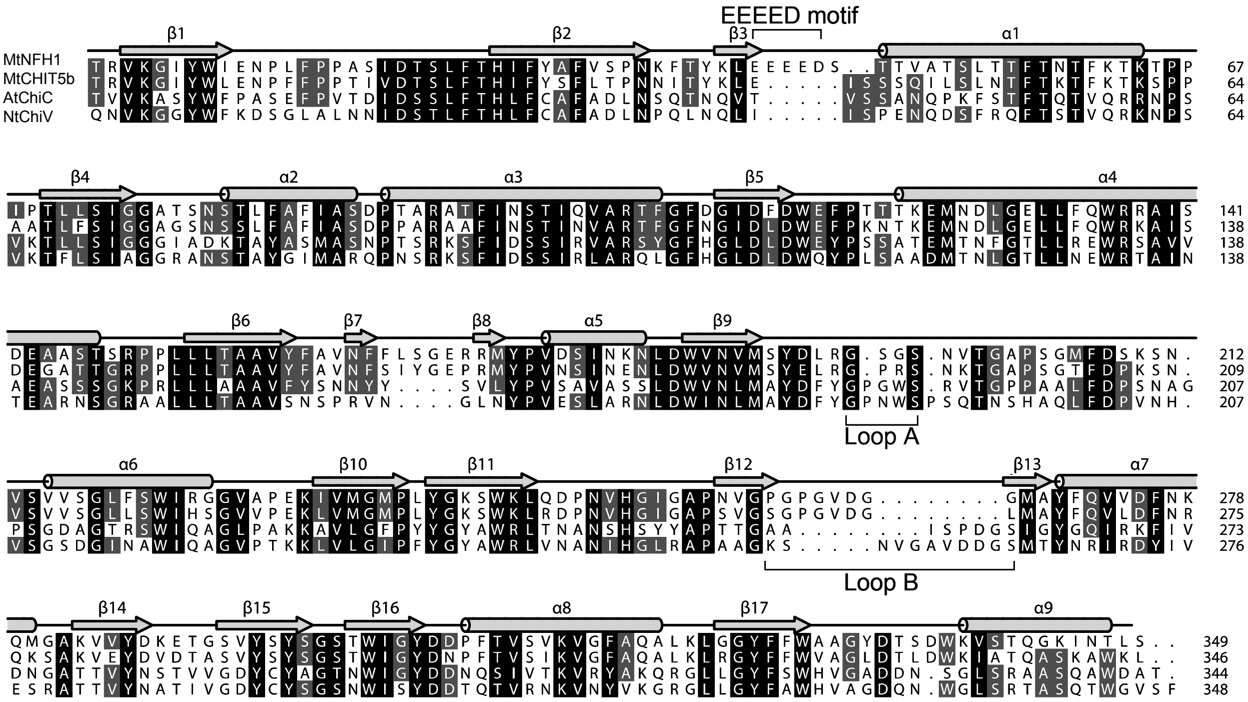

Supplementary Figure S10. Structure-based sequence alignment of MtNFH1 and MtCHIT5b of *M. truncatula* (ecotype R108-1). The previously characterized class V chitinases of *Arabidopsis thaliana* (AtChiC; accession no. BT029539) and *Nicotiana tabacum* (NtChiV; X78325) served as references. The alignment was used for homology modeling in MODELLER. The alignment starts with residue 35 (T) of MtNFH1 (KC833515) and with residue 32 (T) of MtCHIT5b (KU041646). Conserved amino acid residues are highlighted by a black background, and hyphens indicate gaps inserted for optimal alignment of the sequences. The predicted secondary structure of the proteins (α-helices and β-strands) is shown above the sequences. Loop A, loop B and the EEEED motif of MtNFH1 are also marked.

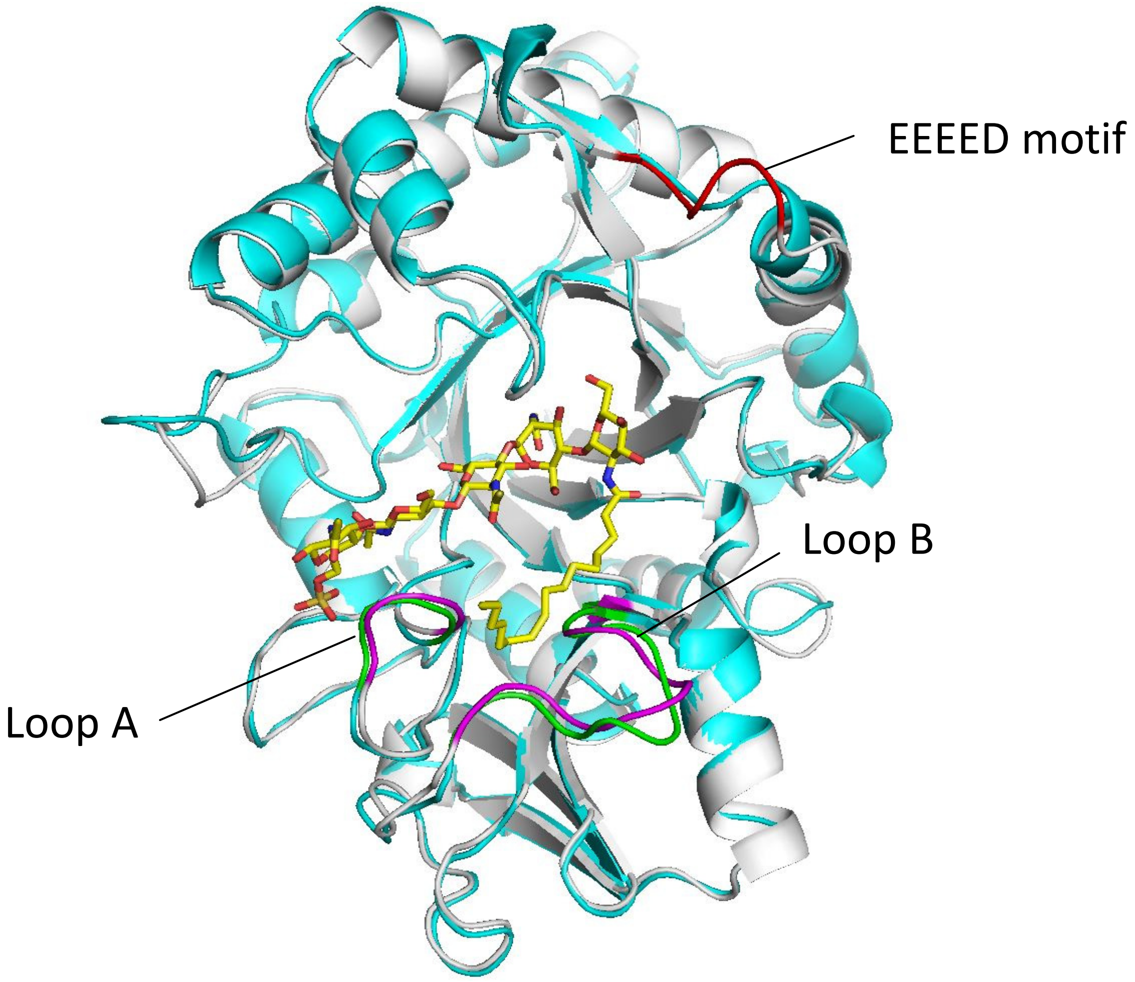

Supplementary Figure S11. The EEEED motif of MtNFH1 is far away from the predicted fatty acid-binding cleft. The predicted structures of MtNFH1 (grey) and MtCHIT5b (cyan) are superimposed to illustrate their similarities. NodSm-V(C16:2, S) was modeled in the binding pocket of MtNFH1. The loops A and B of MtNFH1 are highlighted in magenta and those of MtCHIT5b are green coloured. The EEEED motif is absent in MtCHIT5b.
